# Cranial muscle reconstructions quantify adaptation for high bite forces in Oviraptorosauria

**DOI:** 10.1038/s41598-022-06910-4

**Published:** 2022-02-22

**Authors:** Luke E. Meade, Waisum Ma

**Affiliations:** University of Birmingham, Birmingham, UK

**Keywords:** Palaeoecology, Palaeontology

## Abstract

Oviraptorosaurians are an unusual and probably herbivorous group of theropod dinosaurs that evolved pneumatised crania with robust, toothless jaws, apparently adapted for producing a strong bite. Using 3D retrodeformed skull models of oviraptorid oviraptorosaurians *Citipati*, *Khaan*, and *Conchoraptor*, along with the earliest diverging oviraptorosaurian, *Incisivosaurus*, we digitally reconstruct jaw adductor musculature and estimate bite force to investigate cranial function in each species. We model muscle length change during jaw opening to constrain optimal and maximum gape angles. Results demonstrate oviraptorids were capable of much stronger bite forces than herbivorous theropods among Ornithomimosauria and Therizinosauria, relative to body mass and absolutely. Increased bite forces in oviraptorid oviraptorosaurians compared to the earliest diverging oviraptorosaurian result from expanded muscular space and different cranial geometry, not changes in muscular arrangement. Estimated optimal and maximum possible gapes are much smaller than published estimates for carnivorous theropods, being more similar to the herbivorous therizinosaurian theropod *Erlikosaurus* and modern birds. Restrictive gape and high bite force may represent adaptation towards exploiting tough vegetation, suggesting cranial function and dietary habits differed between oviraptorids and other herbivorous theropods. Differences in the relative strength of jaw adductor muscles between co-occurring oviraptorids may be a factor in niche partitioning, alongside body size.

## Introduction

Oviraptorosaurians are pennaraptoran theropods that include some of the most specialised, aberrant dinosaurs, with the later-diverging members splitting into two major clades—Oviraptoridae and Caenagnathidae^1,2^. The skull morphology of oviraptorid oviraptorosaurians appears to be adapted towards producing a powerful sustained bite^3–6^. Though their crania are heavily pneumatised, they are short and tall, have expanded spaces for jaw musculature, and are equipped with a deep mandible and a robust palate terminating in a toothless beak. This anatomy has been speculated as consistent with forms of durophagy (i.e. egg eating, molluscivory^3,7^) but there is a strong case for the Oviraptoridae being primarily herbivorous^4–6,8–11^ numbering them among a very few herbivorous theropod groups (with ornithomimosaurs and therizinosaurs^10^). We quantitatively assess the functional capabilities of the oviraptorosaurian skull using digital techniques, focussing on their adductor myology, to better understand their jaw function and possible dietary niche.

Digital muscle reconstructions have previously been used to estimate bite forces and make comparisons of adductor muscle anatomy in ornithomimids and therizinosaurs^12,13^, and among other herbivorous dinosaurs (i.e. *Psittacosaurus*^14^; sauropods such as *Camarasaurus*, *Plateosaurus*, and *Diplodocus*^15,16^). In this study, we use computed tomographic (CT) and photogrammetric datasets representing the crania of *Citipati osmolskae*, *Khaan mckennai*, and *Conchoraptor gracilis*, oviraptorid oviraptorosaurians from the Late Cretaceous (Campanian) of Mongolia^5,17–21^. We also study the earliest diverging oviraptorosaurian *Incisivosaurus gauthieri*, from the Early Cretaceous of China (Barremian)^22^. *Incisivosaurus* is one of the very few oviraptorosaurians with teeth (along with caudipterids), which bear wear facets that are a strong indicator of herbivory^22^. We use the CT and photogrammetric data to create retrodeformed 3D models of these species’ crania and mandibles. Based on the retrodeformed 3D models, we reconstruct oviraptorosaurian cranial adductor musculature and use the reconstructions to estimate bite forces. Additionally, we assess how the reconstructed adductor muscle anatomy may have constrained the maximal angle of gape in each species^23^.

This set of four 3D skull models and myological reconstructions allows us to compare bite forces, adductor muscle anatomy, and jaw function between the earliest diverging oviraptorosaurian and later diverging oviraptorids, and between oviraptorosaurians and other herbivorous theropods. Our results are of interest for the question of if and how diet changed with cranial function over the course of oviraptorid evolution.

## Institutional abbreviations

IVPP: Institute of Vertebrate Paleontology and Paleoanthropology, Chinese Academy of Sciences, Beijing, China; MPC: Mongolian Palaeontological Centre, Ulaanbaatar, Mongolia; PIN: Paleontological Institute, Russian Academy of Sciences, Moscow, Russia; ZPAL: Institute of Paleobiology, Polish Academy of Sciences, Warsaw, Poland.

## Methods

### Digitisation and retrodeformation of specimens

CT scans of the crania of *Incisivosaurus* (IVPP V13326), *Citipati* (MPC-D 100/798), *Khaan* (MPC-D 100/973; also including mandible), and *Conchoraptor* (MPC-D 100/3006) were provided by A. M. Balanoff (see^24–29^). Photogrammetry was used to digitise mandibular material from *Incisivosaurus* (IVPP V13326), *Citipati* sp. (MPC-D 100/42) and additional partial cranial and mandibular material of *Conchoraptor* (ZPAL Mg-D I/95). CT scanning parameters, photogrammetric methods, and additional information on specimen provenance are summarised in SI 1. CT datasets were segmented in Avizo Lite (version 9.3.0). Digital retrodeformation of the crania and mandibles was based on Lautenschlager^30^ and was performed in Avizo Lite, Blender (version 2.9.0), and Landmark, restoring taphonomic damage as objectively as possible. This involved interpolation of material over cracks and breaks, repositioning of disarticulated and fragmented elements, replacement of missing elements by mirroring or modification from related species, and the correction of plastic deformation such as compression and shear. Full information on retrodeformational procedure by specimen is also given in SI 1.

### Volumetric muscle reconstruction

The origin and insertion sites of eight cranial muscles were identified based on skull morphology, studies of related theropod groups, and extant analogues^31^. The methodology for 3D reconstruction of cranial myology was derived from Lautenschlager^12^. The skull geometry of oviraptorosaurians, particularly their large orbit (and likely large eyeball), indicates the origin-insertion path of many cranial muscles cannot be straight and is obfuscated by other structures and each other. We therefore deviated slightly from Lautenschlager^12^ and others^13,15^ in our method by connecting identified origin and insertion sites with simple curves rather than straight cylinders/rods; this also allowed easier modelling of the wrapping of the *m. pterygoideus ventralis*. Bundles of eight Bezier curves were created in Blender between origin and insertion sites of each muscle, following their likely path and avoiding intersections with bone and other muscles. A spherical mesh was created centrally in the orbit and scaled until it contacted the orbit; it was then scaled to 95% of this size (leaving a small presumed space for other tissues and muscles; the eyeball would not contact surrounding bone) to form a basic eyeball that the muscle paths were not allowed to intersect. A default Blender ‘UV sphere’ mesh was subdivided (‘subdivision surface’ modifier; subdivisions 2) and shrinkwrapped (‘shrinkwrap’ modifier) around each bundle of curves to form a convex hull. These simple volumes were smoothed and remeshed. Minor areas of overlap occurred between convex hulls in crowded regions where multiple muscles met their origin/insertion sites. Rather than removing overlaps in Blender (i.e. using additional editing or Boolean modifiers), the Blender muscle volumes were imported into Avizo to flesh them out in the same way as Lautenschlager^12^ and others^13,15^ and overlapping volumes were resolved by allocating half the overlap to each muscle, or making corrections where a muscle volume was erroneously encroaching on another’s defined origin/insertion site. The basic muscle volumes were expanded equally in the Avizo segmentation editor (‘grow selection’) until they touched each other and were constrained by osteology, reaching their limits. These were then smoothed in Avizo’s segmentation editor to form the final reconstructions.

Muscle force estimates were calculated following the dry skull method ^32^. Values for muscle cross-sectional areas (CSA) were calculated by dividing muscle volume (given by Avizo surface area and volume module) by its length (obtained by Avizo measurement module). The CSA of each muscle was multiplied by an assumed isometric muscle stress value of 0.3 N/mm^2^^32–34^.

$$Fmus = CSA \times \sigma$$

Calculated muscle force values were multiplied by a correction factor of 1.5 following Thomason ^32^ to account for underestimation due to factors such as muscle pennation not being accounted for. Muscle forces and derived bite forces are reported in this study with this correction factor; SI 2 also gives full sets of values without the correction factor. To calculate the resultant vertical force vectors acting at muscle attachments points on the mandible, muscle forces were multiplied by the cosines of the insertion angles of muscles, measured (Avizo measurement module) in the sagittal ($\alpha$) and coronal ($\beta$) planes on the 3D reconstruction.

$$Fres = Fmus \times cos\alpha \times cos\beta$$

Contribution toward bite force from each muscle was estimated at three points on the palate of each species: the anterior tip of the beak/teeth; the middle level of the palate/toothrow; the tooth-like projection in the posterior of the oviraptorid palate/the posteriormost teeth, to assess a complete range of positions anteroposteriorly that may be contacting food. Estimates of bite force were calculated by rearranging the relationship between outlever length (distance from bite point to jaw joint) and the inlever length (distance from insertion point of muscle to jaw joint). Bite forces calculated for each side of the mandible were summed for the final total bite force estimates.

$$Fbite{ } = { }\left( {Fres{ } \times { }Linlever} \right){ } \div { }Loutlever$$

We assume all adductor muscles participated equally and fully during contraction.

### Musculoskeletal constraints on gape angle

The retrodeformed cranium and mandible models were imported into Blender for muscle-constrained gape analysis following Lautenschlager^12^. The separate cranium and mandible components were connected using an armature (of two bone elements) with a centre of rotation at the jaw joint. Blender’s keyframe animation tool was used to animate and model a jaw opening cycle in which one frame represented 0.5°. The jaw adductor muscles were modelled as two simple cylinders connecting the anteriormost and posteriormost extent of the muscle’s origin and insertion sites. Curved connections between muscle origin and insertion (as used for the basis of our anatomical muscle reconstruction) were not modelled for this analysis; they only minimally affected estimates of optimal and maximum gape angle and their stretch during jaw opening was too uncertain to model objectively. The cylinders were connected to the armature, allowing them to extend as the mandible rotated. A python script (adapted from that of^23^) was used to measure the strain of each muscle cylinder throughout the modelled jaw opening cycle and export the values to a text file.

Muscles comprise a structure of overlapping filament cross-bridges and inherently have a strain range over which maximal tetanic contraction can be achieved (optimal tension up to 130% of resting length) and a maximum tension limit (170% of resting length^35,36^). This structural constraint was used by Lautenschlager^23^ to estimate the gape angle at which the limit of optimal tension is reached and the maximum limit of gape that might occur for muscle tension to still be possible. The optimal and maximum limits of gape were therefore estimated once a muscle cylinder reached 130% and 170% resting length respectively. The script and Blender setup could be set to terminate the cycle when a muscle cylinder reached a determined strain ratio between its stretched and relaxed state and render this terminal step in the jaw opening cycle.

The resting gape must lie at a small open angle, given the length-tension relationship of muscles, in order to generate necessary force during biting^35,37^. Lautenschlager tested theropod skulls at resting gape angles of 3° and 6°, concluding these to approach realistic values. The oviraptorosaurian models here were tested from a resting gape of 5°; this was the degree of gape at which the 3D anatomical reconstruction of the jaw muscles was done.

## Results

### Cranial myology

**Figure 1:**
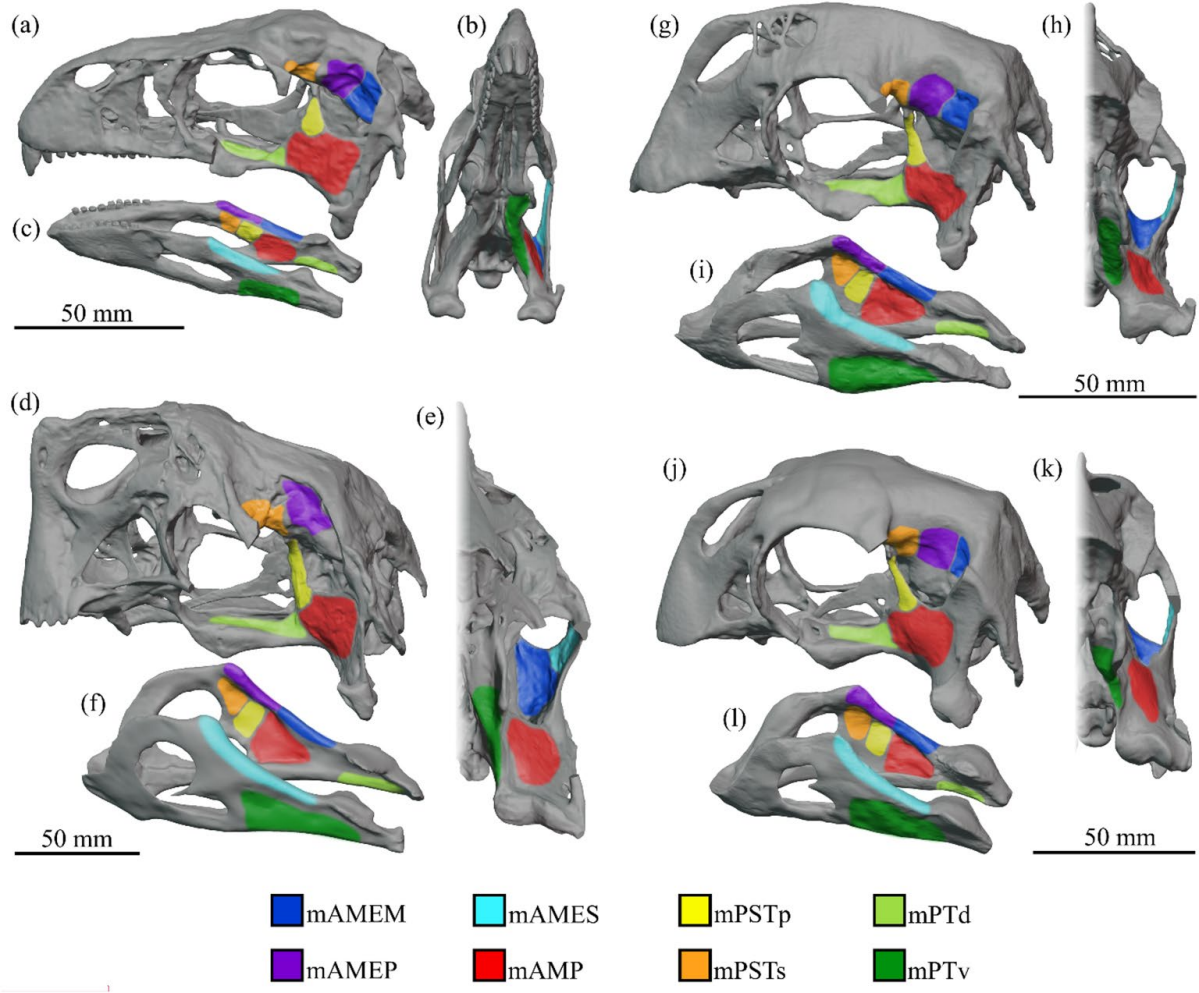
Locations of reconstructed jaw adductor muscle origin and insertion sites for *Incisivosaurus gauthieri* (**a**-**c**), *Citipati osmolskae* (**d**-**f**), *Khaan mckennai* (**g**-**i**), and *Conchoraptor gracilis* (**j**-**l**). Crania are shown in dorsolateral view (**a**,**d**,**g**,**j**) with temporal and postorbital bars removed to better show medial regions within supratemporal fenestra. The left sides of the crania are shown in anteroventral view (**b**,**e**,**h**,**k**) with lower temporal and postorbital bars removed to better show posterior and lateral regions within supratemporal fenestra. Mandibles shown in dorsolateral view (**c**,**f**,**i**,**l**), lateral muscle insertions sites are shown on the left rami, medial insertion sites on the right rami. Scale bars 50 mm. Muscle abbreviations given in results section.

**Figure 2:**
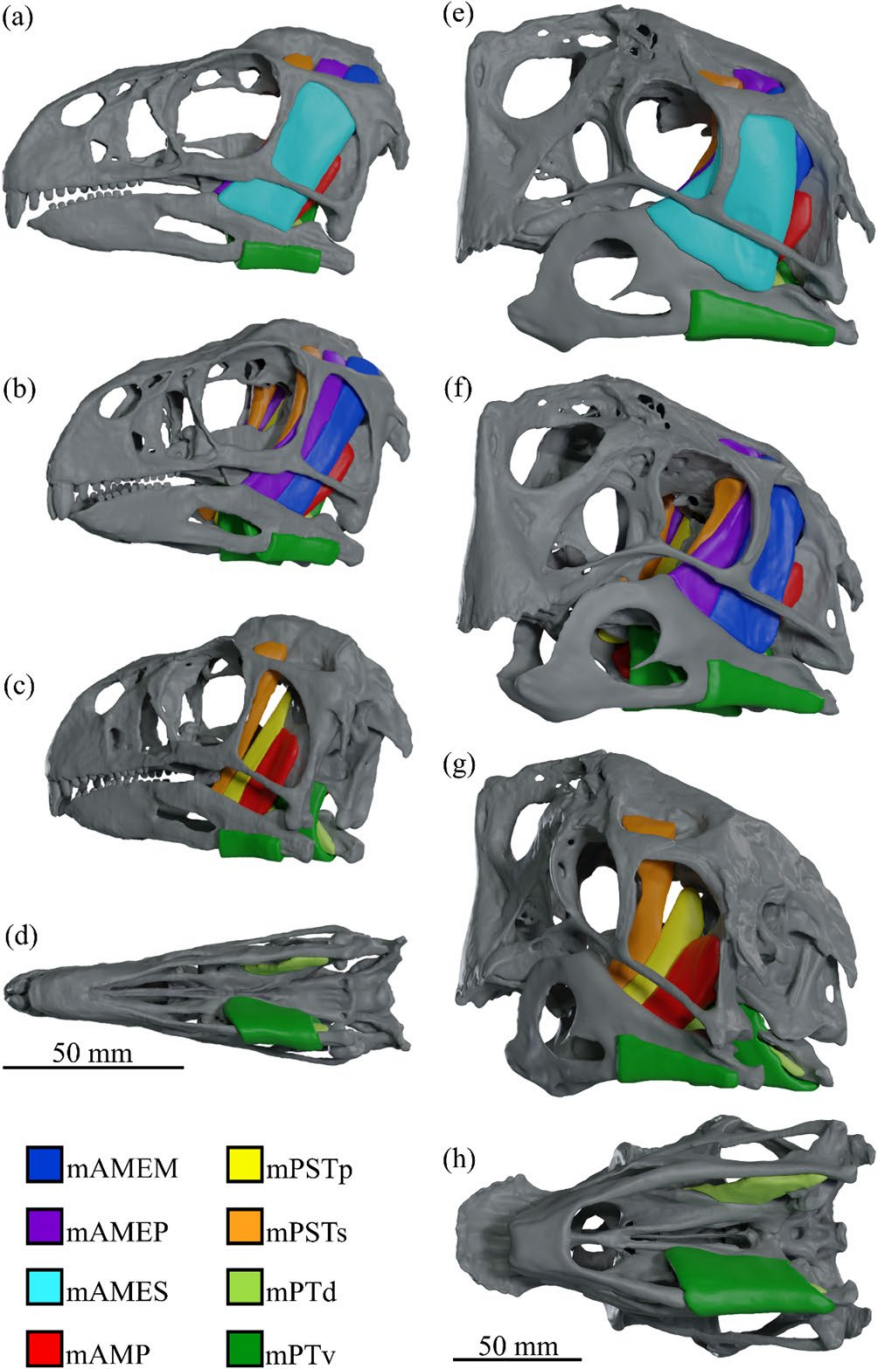
Reconstructed jaw adductor musculature of *Incisivosaurus gauthieri* (**a**-**d**) and *Citipati osmolskae* (**e**–**h**) shown complete in lateral view (**a**,**e**), anterolateral view with mAMES removed (**b**,**f**), posterolateral view with mAME complex removed (**c**,**g**), and ventral view with only the mPT muscles (mPTv removed on left). Scale bars 50 mm, legend colour coded to identify individual muscles. Muscle abbreviations given in results section.

**Figure 3:**
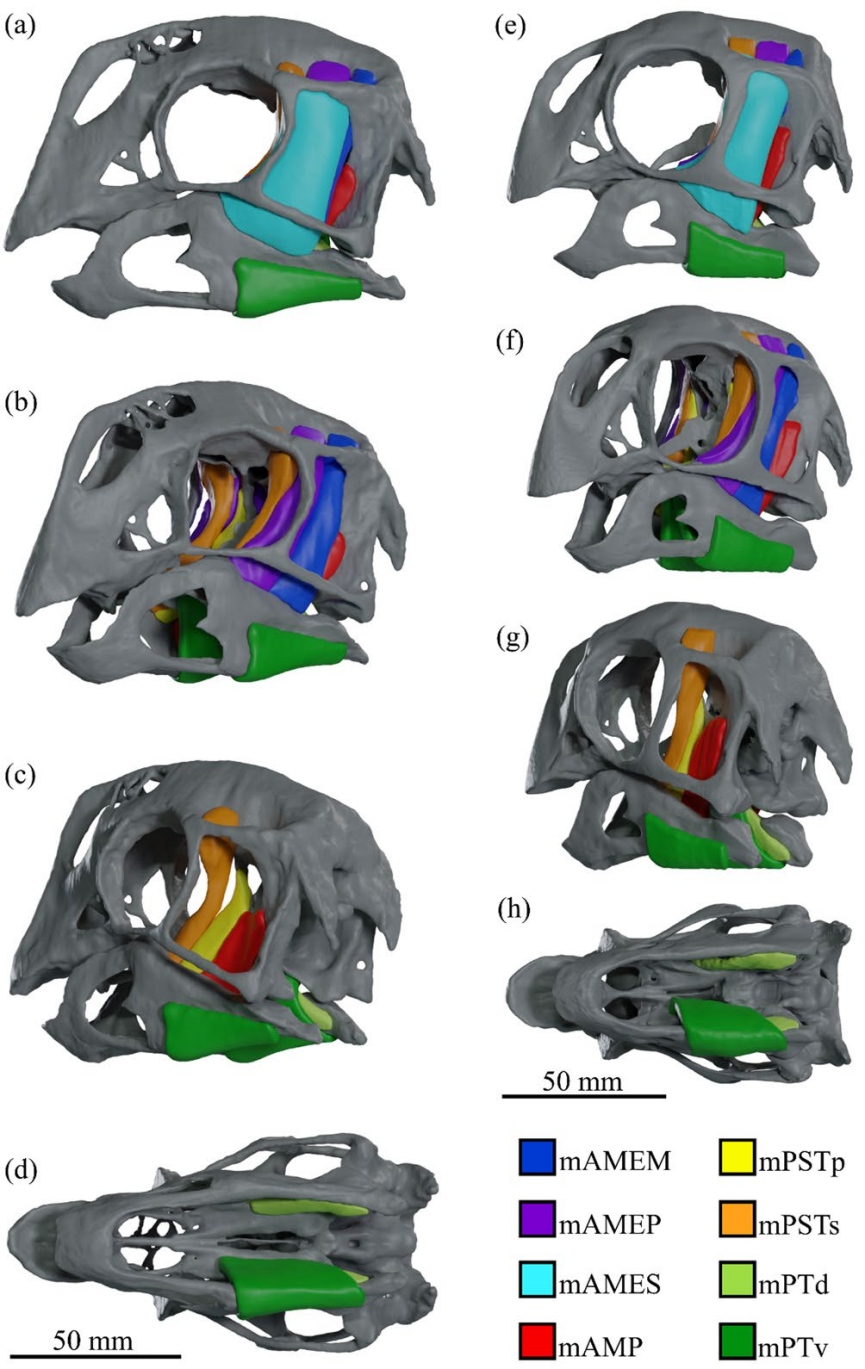
Reconstructed jaw adductor musculature of *Khaan mckennai* (**a**-**d**) and *Conchoraptor gracilis* (**e**–**h**) shown complete in lateral view (**a**,**e**), anterolateral view with mAMES removed (**b**,**f**), posterolateral view with mAME complex removed (**c**,**g**), and ventral view with only the mPT muscles (mPTv removed on left). Scale bars 50 mm, legend colour coded to identify individual muscles. Muscle abbreviations given in results section.

The muscular origin and insertion sites interpreted in the cranium and mandible of each species are identified in Fig. 1; the 3D reconstructed cranial adductor muscles are shown in Fig. 2 (*Incisivosaurus* and *Citipati*) and Fig. 3 (*Khaan* and *Conchoraptor*).

### m. adductor mandibulae externus medialis (mAMEM)

The origin site of the mAMEM is less clear than others of the mAME group^31^ and we reconstruct it, as others have done, in the posterior portion of the supratemporal fossa^12,13,16^ where it is constrained anterolaterally and anteromedially by the positions of mAMES and mAMEP (Fig. 1). This region comprises parts of the squamosal and parietal in all four taxa and is generally vertical, concave, and featureless in all apart from *Citipati.* In this taxon, within the supratemporal fossa, the squamosals and parietals are flattened and orientated to form a deep and concave platform directly perpendicular to the line of action of this muscle (Fig. 1e). The extent and direction of the mAMEM body are somewhat constrained in all taxa by the anterior, dorsal, and posterior edges of the squamosal, quadrate flange, and epipterygoid respectively.

The insertion sites are typically unclear^12,31^. The surangular dorsomedially forms a shelf that overhangs the adductor fossa in *Citipati*, *Khaan*, and *Conchoraptor* (potentially taphonomically exaggerated in the latter two). Insertion onto the dorsomedial and posterior margin of the coronoid eminence (along with insertion of the mAMEP onto the eminence) has been suggested for the mAMEM^12,13,31,38^, but the palatal morphology (especially in the oviraptorids) restricts space around the coronoid eminence so that we do not reconstruct both the mAMEM and mAMEP as inserting in this area. Instead, we reconstruct the mAMEM as inserting on the shelf-like upper part of the surangular’s dorsomedial surface, posterior to the more anterior insertion of the mAMEP, allocating roughly half of the available surface to each (Fig. 1c,f,i,l). This insertion surface is unclear and largely reconstructed in *Incisivosaurus* where there is less well-defined slight convexity on the upper part of the medial surangular surface (Fig. 1c). This area of the retrodeformed mandible model for *Conchoraptor* uses material from *Khaan* and the two are thus similar (Fig. 1i,l).

It is possible the mAMEM and mAMEP merged along their path or did indeed both insert in relation to the coronoid eminence^39^ but ultimately this would not change reconstructed bite force results significantly.

### m. adductor mandibulae externus profundus (mAMEP)

The mAMEP generally has a medial and/or anteromedial origin within the supratemporal fenestra. A vertical crest, similar to that interpreted as the anterior border of the origination site in *Carcharodontosaurus* and *Daspletosaurus*^31^, *Allosaurus*^40^, *Corythosaurus*^41^, and *Erlikosaurus*^12^, is also identified in *Citipati*^19^ (Fig. 1d). We interpret it as the boundary between the mAMEP and mPSTs origins. A small sharp prominence, perhaps similar, is present on the lateral surface of the braincase in *Incisivosaurus* (Fig. 1a). The surface is more featureless in *Khaan* and *Conchoraptor* (Fig. 1g,j), so the anterior limit of the mAMEP origin is constrained by the origin area of the mPSTs (in turn based on the extent and position of the laterosphenoid).

In *Citipati*, a pneumatic opening in the posterolateral wall of the parietal (visible at the posterior of the mAMEP origin in Fig. 1d), underneath where the squamosal contacts the parietal to form the posteromedial margins of the supratemporal fenestra, seems to limit the mAMEP origin posteriorly, dividing it from the mAMEM. A similar opening is not as large or obvious in the other taxa, but similar limits to the origination sites are constrained by the geometry of the supratemporal fenestra. The dorsal extent of the origin is also clear in *Citipati* where a sharp lateral edge, running from the frontal-parietal contact posterolaterally to form the posterior boundary of the supratemporal fossa, separates the dorsal surface of the parietals from their lateral surfaces that contribute to the supratemporal fossa (Fig. 1d). This edge may function for muscle attachment similarly as suggested for a parietal ridge in the oviraptorid *Osoko*^2^.

We reconstruct the mAMEP inserting more anteriorly than mAMEM on the mandible (Fig. 1c,f,i,l), including around the apex of the coronoid elevation itself, along with the mAMES, specifically on the dorsomedial surface of coronoid prominence^31,39^.

### m. adductor mandibulae externus superficialis (mAMES)

In all taxa, the mAMES can be reliably hypothesised to originate on the supratemporal bar^31^ (Fig. 1b,e,h,k). In oviraptorosaurs, this is formed by the postorbital and squamosal. The supratemporal bars in all taxa are mediolaterally flattened, with the medial surface directed slightly ventromedially, more so in *Citipati* than the others (Fig. 1e). The postorbital bars are concave along almost the entire medial surface in *Citipati*. In the other taxa, only the squamosal contribution is concave, with the postorbital ramus being flat or perhaps weakly convex in *Khaan* (Fig. 1h). There are no clear osteological signs of the extent of the mAMES origin site so we restrict it to the medial surfaces of the supratemporal bar as the ventral surface is narrow (as the bars are mediolaterally thin) and the medial surface is slightly orientated in the correct muscle direction in all taxa. The mAMES is reconstructed as originating along the full extent of this medial surface with its anterior and posterior limits constrained by the origins of the mPSTs and mAMEM respectively.

The main body of the jugal has a trough-like gently concave medial surface in all taxa (especially so in *Conchoraptor* where the postorbital process of the jugal also has confluent concavity on its posteromedial surface) that appears like its form would neatly wrap over the exterior of the mAMES as it bulged outwards laterally and followed it anteroventrally on its origin-insertion path.

The mAMES likely inserts onto the dorsolateral edge and lateral surface of the surangular^31,39^, on a shelf running from the coronoid process to the articular (Fig. 1c,f,i,l). This shelf is more strongly defined in the later diverging taxa, especially *Citipati* (Fig. 1f) and *Khaan* (Fig. 1i). The mandibles of the oviraptorids bear apically triangular coronoid eminences, which are anteriorly displaced compared to those of other herbivorous dinosaurs. This has been hypothesized to increase mechanical advantage and attachment area for the temporal musculature as an adaptation for a stronger crushing bite^3,8,38^. The anteriorly displaced coronoid eminence in oviraptorids has been hypothesized to indicate a more anteriorly extending mAMES (as suggested for some ornithischians^39,42^). The mAMES is reconstructed thus here. The insertion site is constrained ventrally by the reconstructed extent of the mPTv insertion site, and dorsomedially by the insertions of the mAMEM and mAMEP, which insert onto the dorsomedial surface of the surangular.

### m. adductor mandibulae posterior (mAMP)

The mAMP is a well-constrained muscle of the adductor chamber, consistently attaching to the lateral surface of the quadrate in an extant phylogenetic bracket^31^. We reconstruct the origin site as the lateral surface of the pterygoid flange of the quadrate (Fig. 1), covering most of this broad flat wing but not encroaching on the epipterygoid (where the mPSTp is present) and pterygoids (where the mPTd originates). No clear muscle scar is apparent in any of the studied taxa. The mAMP origin may also have extended posterodorsally onto the confluent lateral surface of the squamosal, where a curved ridge may demark an expanded origin site for the mAMP in *Conchoraptor* (Fig. 1j)^5^; *Khaan* has a similar morphology (Fig. 1g). This expansion is not reconstructed in earnest—the organization of the other muscle volumes, particularly the passage of the mAMEM, would only permit a thin sliver of extra volume to be created on the expanded origin site, not significantly increasing overall volume, direction, or morphology of the mAMP.

The mAMP inserts in the adductor fossa on the medial mandibular surface (Fig. 1c,f,i,l), occupying most of its main extent and posterior and ventral margins^31^. The adductor fossa in the oviraptorids is large and anteriorly displaced^19,38,39^ and much more significant than that of *Incisivosaurus* (Fig. 1c).

### m. pseudotemporalis superficialis (mPSTs)

In all four taxa, the mPSTs originates on the anterior and/or anteromedial wall of the supratemporal fenestra. In *Citipati*, the area is formed predominantly by the capitate process of the laterosphenoid and the posterior portions of the frontal (Fig. 1d). This surface is concave and rugose. The lateral surface of the laterosphenoid is also rugose, indicating a muscle attachment^19^. The site is bounded laterally by the postorbital, and two ridges may constrain the origin site of the mPSTs ^19^: a sharp ridge runs posteromedially from the capitate process of the laterosphenoid to the epipterygoid contact, forming the ventral boundary, and a vertical ridge on the medial wall of the supratemporal fossa constrains the origin posteromedially, demarking it from the mAMEM. A triangular anterodorsal-posteroventral sloping surface (where a clear frontoparietal fossa has been lost in derived oviraptorids) extends to the dorsotemporal fossa. The anterodorsal extent of the mPSTs origin site on this surface is unclear. The frontoparietal fossa has been argued as a vascular space in dinosaurs rather than a site of muscle attachment^43^, and we place the mPSTs similarly (^43^; Fig. 7 therein), extending into this sloping triangular space but not wholly filling it. We do not reconstruct any attachment of the mPSTs extending onto the frontal processes of the postorbitals.

In *Khaan*, the origin site is less well preserved (Fig. 1g). The mPSTs origin is placed in a similar position to *Citipati* and may extend slightly onto the lateral surface of parietals which contribute to the area. Similarly, in *Conchoraptor* (Fig. 1j), there is more of a contribution of the parietal to the anterior wall of supratemporal fenestra, but very little or no contribution of the frontal. In *Conchoraptor*, the whole origin site is more anteromedially positioned, and exhibits a large smooth exposure of the laterosphenoid. There are no obvious scars or ridges in the above-mentioned area of *Khaan* and *Conchoraptor*. In *Incisivosaurus*, the anterior corner of the supratemporal fossa is narrow and the mPSTs is more anteromedially positioned (Fig. 1a). The origin site likely comprises the laterosphenoid and small parts of the frontal and parietal.

The insertion of the mPSTs is likely related to the medial aspect of the coronoid elevation and parts of the medial adductor chamber^39^. As the medial regions of the coronoid elevation are occupied by the mAMEP in our reconstruction we position the mPSTs, as the deepest temporal muscle, inserting into the anterior portion of the medial mandibular fossa^31^ and its anterodorsal rim (Fig. 1c,f,i,l).

### m. pseudotemporalis profundus (mPSTp)

The mPSTp likely attached to the epipterygoid when present in dinosaurs^31^. When first described in detail, the epipterygoid of *C. osmolskae* (Fig. 1d) was noted as the largest of any known theropod, with a unique strongly twisted body and dorsal tip hosting robust muscle scars^19^. We therefore locate the mPSTp origin site on the epipterygoid of each taxon with confidence and reconstruct its origin along the length of the epipterygoid, which is present in all four taxa (though partially reconstructed in *Khaan* and *Conchoraptor*) (Fig. 1g,j).

The insertion site is problematic but based on extant taxa the muscle likely inserted along the medial surface of the coronoid process or surangular ^31^. As the coronoid process is occupied by the insertions of the mAMES and mAMEP, we position the insertion of the mPSTp dorsomedially on the surangular, occupying the dorsal rim of the mandibular adductor fossa, the position being largely constrained dorsally by the insertions of the mAMEM and mAMEP (Fig. 1c,f,i,l).

### m. pterygoideus dorsalis (mPTd)

The origin site of the mPTd is reconstructed as the linear dorsal surface of the pterygoid in all oviraptorids where a longitudinal concavity runs anteriorly along their length anterior of the pterygoid flange (*Khaan* has a convex dorsal surface but the origin site is modelled similarly (Fig. 1h), and possibly the anteriormost dorsolateral surface of the pterygoid flange. The site is limited anteriorly and anterolaterally by the palatines and ectopterygoids, onto which no attachment was modelled as they are relatively small and delicate. In *Incisivosaurus*, the anterior extent of the origin site is constrained by the level of the jugal ramus of the ectopterygoid anterolaterally and the main body of the ectopterygoid laterally to around a longitudinal concavity on the dorsal surface of the pterygoid (Fig. 1a)—there seems very little/no origination on the palatine.

The mandibular insertion of the mPTd is commonly regarded to be onto the medial surface of the articular and retroarticular process^31^. We reconstruct the mPTd in this position (Fig. 1c,f,i,l), inserting in the narrow medial surface of the posterior aspect of the mandibular ramus, under the medial facet of the articular glenoid and posteriorly onto the medial surface of the retroarticular process.

### m. pterygoideus ventralis (mPTv)

The mPTv is well constrained through phylogenetic bracketing and we reconstruct it in the oviraptorids as originating along the ventral surface of the pterygoid, probably also extending onto the ventral aspect of the pterygoid flange^31^ and posteriorly terminating before the contact with the quadrate. The anterior of the origin is reconstructed as the level of the ectopterygoid contact, with the site entering the longitudinal ventral concavity that is anteriorly confluent with the choanae. In *Citipati*, the pterygoid flange is noted as reduced compared to typically carnivorous theropods, maintaining a roughly consistent width throughout its length (Fig. 1e), as suggested by^19^ to indicate a relatively small m. pterygoideus. However, the main pterygoid body of oviraptorids is relatively elongate. This may be an adaptation to open space for an expanded mAME group to insert onto the mandible, whilst maintaining volume of the mPT. The pterygoids of *Incisivosaurus* are also elongate and reduced in width (Fig. 1b), though not as extreme as in the derived oviraptorids ^24^. The origin of the mPTv on the pterygoid ventral surface is interpreted as running from the posteroventral margin anteriorly into a trough medial to the ectopterygoid, and lateral of a ventral flange termed the accessory ventral flange by Xu et al.^22^, terminating anteriorly before the palatine contact.

In all taxa, the mPTv wraps around the ventral surface of the mandibular rami and inserts on the broad section of the lateral surface of the mandible (Fig. 1c,f,i,l), predominantly comprising the angular.

### Bite force estimates

Measurements of the final volumetric muscle reconstructions are given in Table 1 along with the calculated muscle contraction force, resultant force acting on the mandible, and relative contribution of each muscle. The oviraptorid oviraptorosaurians show greater muscle volumes compared to the earlier diverging *Incisivosaurus*. This is confirmed by greater muscle CSA values relative to cranial surface area in *Citipati* (1.80 × 10^–2^), *Khaan* (1.77 × 10^–2^), and *Conchoraptor* (1.37 × 10^–2^), compared to *Incisivosaurus* (1.21 × 10^–2^). Table 2 shows the inlever and outlever measurements used to calculate bite force resulting from each cranial muscle (and their relative contribution) and the total estimated bite force in each species, for three different bite positions. These range from 349–499 N in *Citipati* down in order of cranial size to 53–83 N in *Incisivosaurus*. Complete calculations and values for Tables 1 and 2 along with measurements for the cranial models are documented in SI 2.

**Table 1:** Geometric measurements of reconstructed muscles and estimated contraction force (Fmus = (volume / length) × 0.3 N/mm^2^ × 1.5^32,34^). Insertion angles of muscles measured in the sagittal ($\alpha$) and coronal ($\beta$) planes used to calculate resultant vertical force acting on mandible ($Fres = Fmus \times cos\alpha \times cos\beta$).

Muscle	Volume (mm^3^)	Length (mm)	Fmus (N)	Contribution (%)	α	β	Fres (N)	Contribution (%)
***Incisivosaurus***								
AMEM	1492.1	43.4	30.9	12.3	27.6	4.4	27.3	12.2
AMEP	1393.8	44.4	28.2	11.2	30.0	4.2	24.4	10.9
AMES	2242.3	41.7	48.4	19.2	24.2	1.5	44.1	19.6
AMP	671.5	21.9	27.7	11.0	27.4	2.1	24.6	10.9
PSTp	379.0	31.1	11.0	4.4	26.6	5.3	9.8	4.4
PSTs	698.1	45.7	13.7	5.5	19.4	1.5	13.0	5.8
PTd	589.3	29.8	17.8	7.1	44.4	11.8	12.5	5.5
PTv	1695.8	20.7	73.7	29.3	13.3	15.6	69.1	30.8
**Sum**	9161.7		251.5				224.7	
***Citipati***								
AMEM	16,130.0	69.2	209.9	15.6	23.8	4.0	191.6	16.6
AMEP	10,122.5	59.9	152.1	11.3	32.4	8.4	127.1	11.0
AMES	19,580.5	58.7	300.2	22.2	16.3	6.8	286.2	24.8
AMP	7221.5	43.8	148.4	11.0	44.7	14.7	102.1	8.8
PSTp	4876.0	50.9	86.2	6.4	33.6	27.2	63.8	5.5
PSTs	6962.5	64.9	96.6	7.2	25.9	5.9	86.5	7.5
PTd	3638.5	42.2	77.6	5.7	37.5	13.3	59.9	5.2
PTv	10,858.0	35.1	278.8	20.7	19.0	25.1	238.8	20.7
**Sum**	79,389.5		1349.9				1155.9	
***Khaan***								
AMEM	4032.0	48.5	74.9	12.1	28.9	1.2	65.6	12.4
AMEP	4398.0	45.8	86.5	13.9	35.5	7.1	69.9	13.2
AMES	6371.5	46.3	123.9	20.0	17.7	9.3	116.4	22.0
AMP	1782.5	27.5	58.3	9.4	40.4	7.7	44.0	8.3
PSTp	1440.0	34.8	37.2	6.0	39.5	22.5	26.6	5.0
PSTs	2260.5	49.3	41.3	6.7	27.0	2.7	36.8	7.0
PTd	1679.5	30.4	49.7	8.0	47.2	12.7	33.0	6.2
PTv	3900.0	23.6	148.9	24.0	14.9	19.2	135.8	25.7
**Sum**	25,864.0		620.7				528.0	
***Conchoraptor***								
AMEM	2141.5	46.9	41.1	9.4	26.9	2.0	36.6	9.3
AMEP	2950.5	44.5	59.7	13.7	33.4	7.0	49.5	12.7
AMES	4071.0	46.1	79.6	18.3	18.8	6.6	74.8	19.1
AMP	1573.0	27.9	50.8	11.7	24.9	1.6	46.0	11.8
PSTp	786.0	31.1	22.7	5.2	17.1	9.8	21.4	5.5
PSTs	1678.5	47.5	31.8	7.3	22.6	4.1	29.3	7.5
PTd	1096.5	34.2	28.8	6.6	38.6	8.0	22.3	5.7
PTv	3528.0	26.4	120.3	27.7	14.0	17.9	111.1	28.4
**Sum**	17,825.0		434.9				391.1

**Table 2:** Bite force estimates (newtons) for each species, calculated (*Fbite* = (*Fres* × *Linlever*) ÷ *Loutlever*) for three points on their primary palate: the anterior tip of the beak/teeth; the middle level of the palate/toothrow; the tooth-like projection in the posterior of the oviraptorid palate/the posteriormost teeth. Percentages in brackets reported next to the bite force estimates for the oviraptorid taxa show how much greater these estimates are compared to values that would be predicted by scaling up the bite force estimates of *Incisivosaurus* by cranial surface area.

	Anterior			Mid-palate			Posterior			Contribution
	Inlever	Outlever	Fbite (N)	Inlever	Outlever	Fbite (N)	Inlever	Outlever	Fbite (N)	(%)
***Incisivosaurus***										
AMEM	19.8	90.4	6.0	19.8	71.6	7.5	19.8	58.1	9.3	11.3
AMEP	30.3	90.4	8.2	30.3	71.6	10.3	30.3	58.1	12.7	15.4
AMES	23.4	90.4	11.4	23.4	71.6	14.4	23.4	58.1	17.8	21.6
AMP	19.9	90.4	5.4	19.9	71.6	6.8	19.9	58.1	8.4	10.2
PSTp	26.0	90.4	2.8	26.0	71.6	3.6	26.0	58.1	4.4	5.3
PSTs	31.5	90.4	4.5	31.5	71.6	5.7	31.5	58.1	7.0	8.5
PTd	9.4	90.4	1.3	9.4	71.6	1.6	9.4	58.1	2.0	2.5
PTv	17.6	90.4	13.5	17.6	71.6	17.0	17.6	58.1	20.9	25.4
**Sum**			**53.0**			**67.0**			**82.5**	
***Citipati***										
AMEM	37.1	142.0	47.1	37.1	122.0	54.9	37.1	99.4	67.4	13.5
AMEP	61.9	142.0	54.2	61.9	122.0	63.1	61.9	99.4	77.5	15.5
AMES	48.0	142.0	94.4	48.0	122.0	109.9	48.0	99.4	134.9	27.0
AMP	47.2	142.0	33.1	47.2	122.0	38.5	47.2	99.4	47.2	9.5
PSTp	56.8	142.0	25.2	56.8	122.0	29.3	56.8	99.4	36.0	7.2
PSTs	66.7	142.0	39.7	66.7	122.0	46.2	66.7	99.4	56.7	11.4
PTd	18.1	142.0	7.3	18.1	122.0	8.5	18.1	99.4	10.5	2.1
PTv	30.6	142.0	48.2	30.6	122.0	56.1	30.6	99.4	68.8	13.8
**Sum**			**349.3** **(84%)**			**406.5** **(69%)**			**499.0** **(69%)**	
***Khaan***										
AMEM	18.6	103.6	11.8	18.6	88.7	13.7	18.6	72.0	16.9	8.6
AMEP	34.3	103.6	23.2	34.3	88.7	27.0	34.3	72.0	33.3	16.9
AMES	27.4	103.6	30.8	27.4	88.7	36.0	27.4	72.0	44.3	22.4
AMP	26.1	103.6	11.1	26.1	88.7	12.9	26.1	72.0	15.9	8.1
PSTp	36.5	103.6	9.4	36.5	88.7	10.9	36.5	72.0	13.5	6.8
PSTs	41.4	103.6	14.7	41.4	88.7	17.1	41.4	72.0	21.1	10.7
PTd	13.4	103.6	4.3	13.4	88.7	5.0	13.4	72.0	6.1	3.1
PTv	24.7	103.6	32.3	24.7	88.7	37.7	24.7	72.0	46.5	23.5
**Sum**			**137.3** **(54%)**			**160.4** **(42%)**			**197.6** **(42%)**	
***Conchoraptor***										
AMEM	16.8	85.8	7.2	16.8	76.9	8.0	16.8	58.3	10.6	6.7
AMEP	29.8	85.8	17.2	29.8	76.9	19.2	29.8	58.3	25.3	16.1
AMES	24.7	85.8	21.5	24.7	76.9	24.0	24.7	58.3	31.6	20.2
AMP	21.1	85.8	11.3	21.1	76.9	12.6	21.1	58.3	16.6	10.6
PSTp	26.5	85.8	6.6	26.5	76.9	7.4	26.5	58.3	9.7	6.2
PSTs	31.6	85.8	10.8	31.6	76.9	12.0	31.6	58.3	15.9	10.1
PTd	13.6	85.8	3.5	13.6	76.9	4.0	13.6	58.3	5.2	3.3
PTv	22.1	85.8	28.6	22.1	76.9	31.9	22.1	58.3	42.0	26.8
**Sum**			**106.7** **(32%)**			**119.0** **(17%)**			**157.0** **(25%)**	

The condition of the oviraptorid oviraptorosaurian skull is characterised by an increased volume for adductor musculature and increased mechanical advantage resulting from anteroposterior shortening, compared with the more conventional theropod skull geometry of the earlier diverging *Incisivosaurus*. Estimated bite forces conserve a greater proportion of the resultant force applied to the mandible (Fbite/Fres) in the oviraptorids compared with *Incisivosaurus*. This results from greater mechanical advantage in the oviraptorids' jaw for all bite positions, though the difference relative to *Incisivosaurus* is greatest anteriorly (see Table 3.) These two factors result in their comparatively stronger estimated bite forces, an increase of 17–84% greater (depending on species and bite position; see Table 2) than would be predicted by scaling by cranial surface area. The increased relative bite force of the oviraptorids is not a result of more beneficial muscle insertion angles; there is no clear difference in the ratio of resultant muscle force acting on the mandible to the actual muscle force produced (Fres/Fmus) between *Incisivosaurus* (0.894) and the three later diverging taxa (*Citipati*, 0.856; *Khaan*, 0.851; *Conchoraptor*, 0.899).

**Table 3:** Mechanical advantage values for the three different positions of the bite force estimates.

	Mechanical advantage		
	Anterior	Mid-palate	Posterior
*Incisivosaurus*	0.236	0.298	0.367
*Citipati*	0.302	0.352	0.432
*Khaan*	0.260	0.304	0.374
*Conchoraptor*	0.273	0.304	0.401

**Figure 4:**
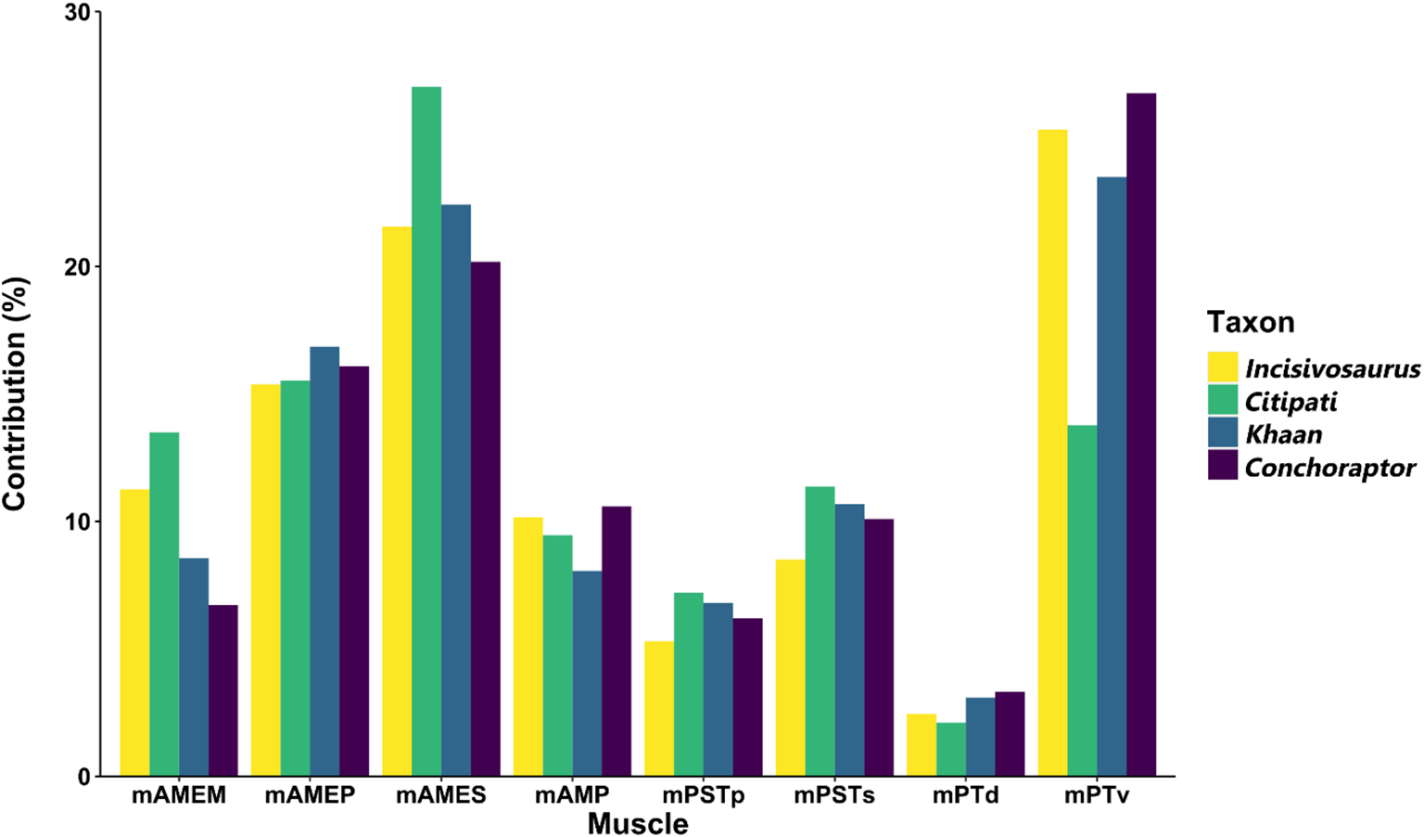
The relative contribution of each cranial muscle to total estimated bite force by species. Note that the condition of *Citipati* appears the most dissimilar to all others in its comparatively stronger mAMEM, mAMES and weaker mPTv.

**Figure 5:**
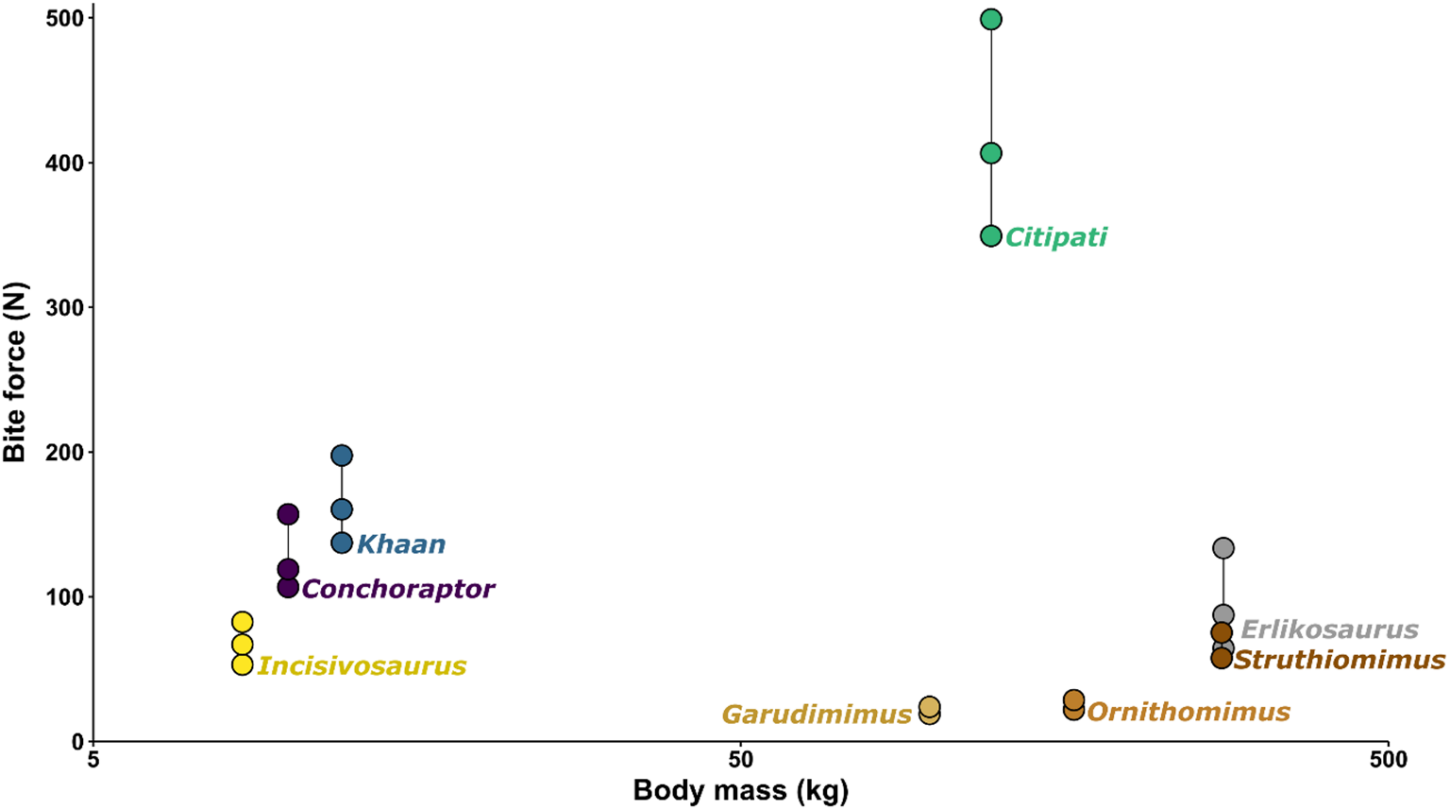
Comparison of the estimated bite forces in multiple positions of *Incisivosaurus* and three oviraptorid oviraptorosaurians with other likely herbivorous theropod taxa that have had estimates made using similar digital volumetric methods^12,13^ show the oviraptorosaurians (oviraptorids especially) are capable of much stronger bite forces both relative to body mass and absolutely. Body mass values from Zanno and Makovicky^11^.

The relative contribution of the different cranial muscles to bite force is broadly similar in each species (Fig. 4). The mPTv is typically the largest component, followed closely by the mAMES, then the rest of the mAME complex. *Citipati* differs from the others with a relatively stronger mAMES and mAMEM, and a relatively low value for the mPTv. The width of the *Citipati* cranium and mandible make the mPTv less vertically orientated and the reconstruction of the mPTv (in all taxa) is less well constrained by bone and other muscle volumes—its volume could be underestimated in all models. No clear difference emerges between *Incisivosaurus* and the later diverging oviraptorids in the relative contributions of cranial muscles to bite, apart from a slightly relatively weaker mPSTp and mPSTs—reconstructed muscles are proportionally similar but relatively larger in the oviraptorids. The bite force estimates of the four oviraptorosaurians (including *Incisivosaurus*) are significantly greater than estimates (from similar digital methods) made for other putatively herbivorous theropods of much larger body mass (Fig. 5) both relatively and absolutely.

### Gape analysis

**Figure 6:**
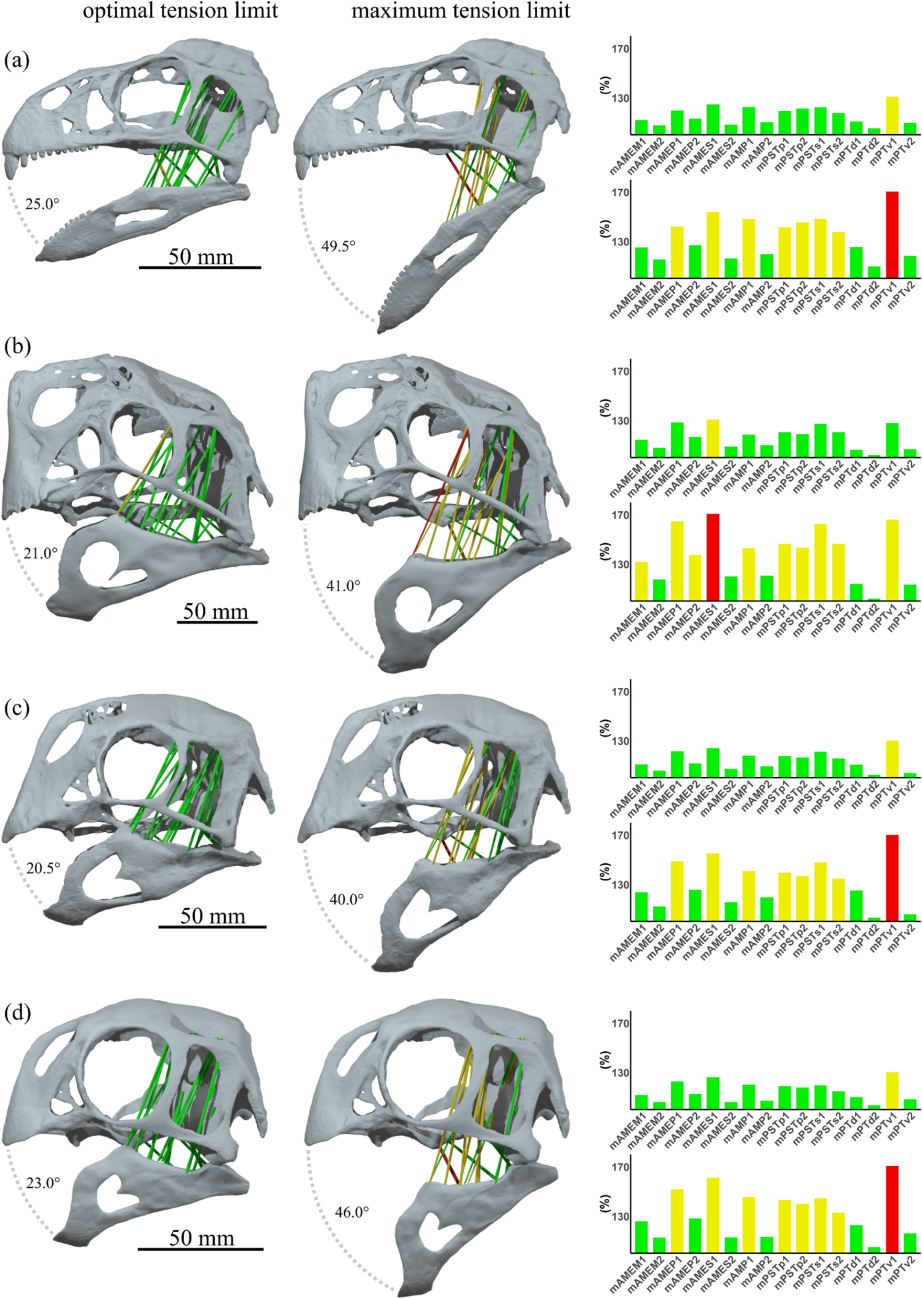
Estimates of the gape angle limit of optimal tension and the maximum limit of gape for muscle tension in *Incisivosaurus gauthieri* (**a**), *Citipati osmolskae* (**b**), *Khaan mckennai* (**c**), and *Conchoraptor gracilis* (**d**) from a muscle resting length at a gape angle of 5°. Bar charts show the strain factors of individual modelled muscle cylinders at optimal and maximum tension limit; anteriormost muscle cylinders suffixed ‘1’, posteriormost suffixed ‘2’. Muscle cylinders (and corresponding bars) are colour coded yellow and red when exceeding 130% and 170% of resting length respectively, otherwise green. Note that the anterior mPTv constrains gape in all species apart from *Citipati* which is constrained by the anterior mAMES. Scale bars 50 mm. Muscle abbreviations given in results section.

The early diverging oviraptorosaurian *Incisivosaurus* showed the highest estimates of optimal (25.0°) and maximum gape limit (49.5°) compared with the oviraptorid oviraptorosaurians, though not by much; estimates for gape limit in *Khaan* were lowest (20.5° and 40.0°), marginally less than *Citipati* (21.0° and 41.0°). Values for *Conchoraptor* (23.0° and 46.0°) lie between *Incisivosaurus* and the others. Figure 6 shows these estimates along with charts of the muscle cylinder strains that they are derived from. The anteriormost cylinder representing the mPTv constrains optimal and maximum gape in all but *Citipati*, in which it is constrained by the anteriormost regions of the mAMES. In this taxon the postorbital half of the skull is particularly low, sloping posteriorly, and the relatively low upper temporal bar directs the strong mAMES ventromedially to a prominent coronoid process of the surangular of the mandible. This leads to a shorter resting length for this muscle, causing its extension during jaw opening to exceed our tension limits just before the mPTv (which is the next most extended). The mAMEM is also relatively more extended in *Citipati*. The other three species are more similar in relative muscular strain, reinforcing the finding that relative muscle strength and arrangement in *Citipati* has more differences compared with other oviraptorids, than between some oviraptorids (*Khaan* and *Conchoraptor*) and earlier diverging oviraptorosaurians (*Incisivosaurus*).

Acting antagonistically to the jaw closing muscles is the m. depressor mandibulae (mDM), primarily responsible for jaw depression (opening). It originates from around the paroccipital processes of the cranium, inserting onto the dorsal aspect of the retroarticular process of the mandible^31,39^. During the gape analysis, we checked mDM length change (from a shorter state at the maximum and optimal estimated gape angles to an elongated state at the 5° resting jaw angle) was not unrealistic. Strain values of the mDM were all calculated to be below the maximum strain limit (1.7) we modelled for the jaw adductors. From its shortest (maximum gape limit) the mDM in *Incisivosaurus* was extended by a factor of 1.08 at the estimated optimal gape limit and 1.20 the 5° resting jaw angle, *Citipati* reached 1.11 and 1.33 respectively, *Khaan* reached 1.16 and 1.48, and *Conchoraptor* reached 1.19 and 1.67.

The oviraptorosaurians show estimated gape limits much lower than those of carnivorous theropods tested by Lautenschlager^12^, more like herbivorous theropod *Erlikosaurus* (optimal tension limit 24.0°; maximum tension limit 49.0°; resting gape of 6°). It is noted that herbivorous species exhibit lower gape angles than carnivorous species^23,44^, and thus our estimates of gape angle may be further support for a herbivorous diet among oviraptorosaurians (when considered against other theropods). Lautenschlager ^12^ notes that experimental results document gape angle in modern birds can reach angles up to around 40°. The maximum gape angles estimated for these oviraptorosaurians are similar to experimental results of gape angle in birds among passerines and Galliformes, which can reach around 40°^45–48^ (though this can be greater in parrots^49^)—a functional similarity between the crania of birds and oviraptorids which, beyond superficial beaked appearance, are quite dissimilar.

## Discussion

This study is the first attempt at quantifying oviraptorosaurian bite forces. Our estimates show the Oviraptoridae were capable of producing greatly stronger bite forces than other herbivorous theropods, and those of *Incisivosaurus* were roughly equal to ornithomimids of body mass roughly 33 times its size. These results suggest that oviraptorid oviraptorosaurians (and to a lesser extent earlier diverging oviraptorosaurians such as *Incisivosaurus*) significantly differed in cranial function from other herbivorous theropod groups of both similar and larger size, potentially feeding on very different foodstuffs. Oviraptorids shared an environment with both ornithomimosaurs and therizinosaurs, and other additional herbivorous dinosaurs such as ankylosaurs, hadrosaurs, and sauropods^5^. The strong bite forces estimated here could have allowed oviraptorids to acquire and process tougher plant material than ornithomimosaurs and therizinosaurs. Herbivores of greatly larger body mass and forms with adaptations towards complex jaw mechanisms or gut processing capabilities (i.e., sauropods, hadrosaurs) could likely also cope with tough vegetation, but oviraptorids may have been able to focus on entirely different food sources purely from the difference in their relative size (focussing on small tough items ignored by larger forms) and height stratification of material (focussing low to the ground). Their jaw strength would also feasibly allow them to handle small prey, to supplement a mostly herbivorous diet, and generally broaden the range of possible food items as a useful tool for both food procurement and initial processing. This adaptability could have given them a competitive advantage among the potentially sparse vegetation of their semi-arid environment^5^.

Suggesting specific food sources is difficult as plant fossils are rare from the formations associated with the oviraptorids studied here^50^. A key dietary focus of oviraptorids may have been small tough stems, nuts or seeds, similar to modern parrots^51^. It is also, however, impossible to satisfactorily compare the two groups as parrot bite forces and body mass differ from those estimated for oviraptorosaurians by an order of magnitude^49^. High bite forces also do not necessitate dietary specialism (i.e. such specificity as molluscivory)—instead, they widen the range of possible food sources with oviraptorosaurians potentially being effective generalists or specialists depending on the environment. The result that *Incisivosaurus* has the lowest jaw mechanical advantage, relative bite force, and highest gape angle may suggest it more retained some plesiomorphic dietary adaptation to omnivory/non-herbivorous foodstuffs. Our gape analysis suggests jaw clearance may limit potential prey items to around 6 cm in maximum transverse dimensions.

The orbits are large in all four oviraptorosaurian species but the shortened crania and more anteriorly positioned coronoid eminence of the mandibles in the oviraptorids result in direct muscle paths between origin and insertion intersecting the presumed space for the eyeball. The muscular reconstructions presented here, with the mAMEP, mAMES, and mPSTs required to curve anteriorly around the presumed ocular space, are reminiscent of a similar muscular condition in parrots^49,52^. We reject any parrot-like muscular attachments onto the jugal^49,52^ as hypothesised in some non-avian dinosaurs^31^ and assessed in the also superficially parrot-like *Psittacosaurus*^14^; the jugals are very thin and delicate in oviraptorids.

Differences in bite force between *Incisivosaurus* and the oviraptorids are chiefly due to different cranial geometries and available space for musculature, rather than changes in muscular arrangement. The steady increase of bite force estimate with size in oviraptorosaurians in this study (shown in Fig. 6) arises from a fairly consistent muscular arrangement in our reconstructions, though there are some differences. The only consistent difference of *Incisivosaurus* compared to the oviraptorids is a relatively slightly weaker mPSTp and mPSTs. The most different in muscle arrangement is *Citipati*, in which the mAMEM and mAMES are relatively stronger than the other oviraptorids and *Incisivosaurus*. The increased relatively contribution of these muscles to bite force in *Citipati* is a result of its morphologically distinctive wide, anterodorsal-posteroventrally sloping occiput. This places the supratemporal fenestra more anteriorly and forms a platform for the mAMEM to be larger and better directed, more efficiently orientating both the mAMEM and mAMES to insert on a relatively larger coronoid process. This is combined with a wider adductor chamber allowing relatively larger musculature, especially expansion of the mAMES which originates from a more robust and concave supratemporal bar with a large amount of space to fill between this origin and its broad insertion on the mandible. *Citipati* shows the largest increase in bite force (relative to cranial surface area, see Table 2) compared with the early diverging *Incisivosaurus* out of the three oviraptorids, and therefore the greatest estimated bite force due to its larger body mass. The increased size and efficiency of the muscles within the adductor chamber in *Citipati*, especially the mAMEM and mAMES, results in the comparatively lower contribution of the mPTv to bite force, with the mPTv also positioned less vertically. However, the extent of the mPTv is more difficult to reconstruct as it is less surrounded by bony constraints both medially and laterally around the mandible and only in substantial contact with the volume of the mPTd. The mPTv volume was reconstructed by growing the basic shrinkwrapped volume to a similar degree as the other muscle volumes were able to be expanded before they were constrained by adjacent muscles, without making the mPTv substantially thicker than its origin area. This resulted in a realistic volume that we estimate as relative strong but could feasibly have been even larger in size.

Oviraptorids (and caenagnathids) have a craniomandibular joint which would have allowed anteroposterior sliding^6,31,38,39^. For anteroposterior movement of the mandible to take place, the origin and insertion of the jaw adductor muscles must off a vertical line^39,53^. Most adductor muscles (mAME and mPST groups) have a posterodorsal line of action. The condition in *Citipati* differs from the other oviraptorids as the mAME group, which contributes the most to bite force, is more vertically orientated and the only muscles positioned off the vertical in an opposing anterodorsal line, the mPT group, are relatively much weaker than the other oviraptorids. This positions the mAME less for palinal motion of the jaw and the mPT would produce a weaker returning forward motion, potentially indicating *Citipati* had a stronger vertical crushing bite with less emphasis on anteroposterior grinding jaw movement. In addition to body size, this could hint at an element of niche partitioning resulting from jaw function between *Citipati* and co-occurring oviraptorids like *Khaan*.

No crania of the other key group within Oviraptorosauria, the Caenagnathidae, are well enough preserved to undertake the same kind of digital myological and biomechanical analyses possible for the species studied here. Essentially no material representing the muscle origin sites identified in this study has been described for caenagnathids but there is a good availability of mandibular specimens^38,53–56^. Caenagnathid mandibles are typically elongate and slender compared to oviraptorid mandibles. The surangular and angular of caenagnathids are less tall and surround a large external mandibular fenestra which is less anteriorly positioned, providing relatively less available space for musculature to insert. Any coronoid eminence is low or absent, presumably reducing the mechanical advantage of the mAME and mPST muscle groups in a condition contrary to the dorsally projecting coronoid eminence of the oviraptorids which increases mechanical advantage. Nevertheless, the adductor musculature of the caenagnathids mandible likely inserted onto similar positions (^39^; Figure 4L of^31^) as oviraptorids. The recognition of a lateral flange on the dentary of *Anzu* and *Gigantoraptor*^55^ has been compared with a similar feature interpreted as an adductor insertion site in dicynodonts^57^ (which have mandibles similar to oviraptorosaurians, capable of anteroposterior sliding movement) but a similar attachment site laterally onto the dentary of caenagnathids would position the mAMES insertion much more anteriorly than that reconstructed here in oviraptorids, likely unrealistically forward relative to the orbit and adductor chamber.

The diet of caenagnathids has been suggested to be more carnivorous than oviraptorids ^6,10,56^. Several caenagnathids mandibles show a sharp, upturned tip and the lower mechanical advantage of their jaws would result in a weaker but quicker jaw opening/closure compared to oviraptorids, a possible adaption for catching mobile prey of a small body size as part of a carnivorous or omnivorous diet^6,10,56^. Herbivory focussing on softer plant material than those consumed by oviraptorids^58^ has also been suggested though caenagnathids appear to lack features positively adapted towards herbivory. However, exceptions such as the huge *Gigantoraptor*, the mandible of which appears short and deep as in those of oviraptorids (the cranium is unknown)^38^, imply a mix of feeding styles and niche partitioning within Caenagnathidae, with adaption among some caenagnathids towards high bite forces as in Oviraptoridae. Lack of material makes clear statements difficult.

It is worth noting that oviraptorid dinosaurs were toothless and likely possessed keratinous beaks. The morphology of rhamphothecae would affect our estimates of bite force, changing bite position and mechanical advantage. The premaxilla and dentary shape of Oviraptoridae is variable and beak shapes within the group are also likely to have varied. However, it is uncertain how closely the rhamphotheca would have followed underlying bone and reconstruction of this covering’s morphology is problematic.

## Conclusion

Muscular reconstructions show oviraptorosaurian dinosaurs were capable of producing relatively strong bite forces, potentially being predominantly herbivorous generalists or specialists depending on the environment. Cranial shortening and expansion of muscle space in oviraptorids increased bite force compared with early diverging oviraptorosaurians, but muscular arrangement remained fairly conservative, differing more within Oviraptoridae itself (*Citipati* differed more from its fellow oviraptorids in the relative contribution of different muscles to bite force than did *Incisivosaurus*).

Our results suggest herbivorous theropods (including oviraptorids) were niche partitioned by both body size, but also clearly by cranial function. Bite forces vary greatly more between oviraptorids and therizinosaurs (and ornithomimosaurs) than do estimated gape limits, and thus were likely the more important niche partitioning component of cranial function.

These results will serve as an ideal stepping off point for further investigation into the cranial functional morphology of oviraptorosaurians, using the retrodeformed specimens and reconstructed muscle force vectors to inform finite element analyses to compare patterns of stress and strain. These studies are ongoing and should reveal more information about the specific ways oviraptorosaurian crania were adapted to utilise their relatively strong bite.

## Supplementary Information

Supplementary Information 1.

Supplementary Information 2.

## Data Availability

The datasets, including 3D models in the format of Blender projects for the retrodeformations, muscular reconstructions, and gape analyses, along with associated python scripts, generated and analysed during the current study are available from the Zenodo data repository and available for download at: https://doi.org/10.5281/zenodo.5585305.
