# Supplementary material for: Cranial muscle reconstructions quantify adaptation for high bite forces in Oviraptorosauria
Source: Sci Rep. 2022 Feb 22;12:3010. doi: 10.1038/s41598-022-06910-4 (PMC8863891; doi:10.1038/s41598-022-06910-4)
Supplement: Supplementary file 1 — Supplementary Information 1. [file 41598_2022_6910_MOESM1_ESM.docx]

**Specimen information and associated retrodeformation procedure**

Luke E. Meade^1^* & Waisum Ma^1^

^1^University of Birmingham

*Corresponding author (luke.edward.meade@gmail.com)

Institutional abbreviations:

**IVPP**: Institute of Vertebrate Paleontology and Paleoanthropology, Chinese Academy of Sciences, Beijing, China; **MPC**: Mongolian Palaeontological Centre, Ulaanbaatar, Mongolia; **PIN**: Paleontological Institute, Russian Academy of Sciences, Moscow, Russia; **STM**: Shandong Tianyu Museum of Nature, Pingyi, China; **ZPAL**: Institute of Paleobiology, Polish Academy of Sciences, Warsaw, Poland.

**1. *Incisivosaurus gauthieri* IVPP V 13326**

*Provenance*

The specimen was collected from the Lujiatun Unit of the lowermost Yixian Formation, near the Luijitun village of Beipiao City, Liaoning, China. These strata date to the Early Cretaceous (Barremian-Aptian; 125.9 Ma in Chang *et al*., 2017; older than 128 Ma in Swisher *et al*., 2002). The specimen comprises a cranium, anterior mandibles, a postdentary mandible fragment (figured by Xu *et al*. (2002) but potentially lost (Balanoff *et al*., 2009)), and a partial cervical vertebra.

*CT-Scanning*

IVPP V 13326 was scanned at Stony Brook University medical scanning facility in 2004. The cranium was scanned along the coronal axis (parameters unknown) yielding 404 DICOM of 512 x 512 pixel resolution. Slice spacing is 0.31 mm, and x-, y-resolution is 0.188 mm. The CT-dataset was initially used by Balanoff *et al*. (2009) for cranial and endocranial descriptions of *I. gauthieri* (endocranial cast subsequently used in Balanoff *et al*., 2013; Balanoff *et al*., 2014; Balanoff *et al*., 2016; Balanoff *et al*., 2018).

*Condition and taphonomic damage*

The cranium is nearly complete on the right side and very incomplete on the left. Much of the cranial interior is still enclosed in matrix as removal would endanger the specimen (Balanoff *et al*., 2009). The cranial elements and teeth present are relatively well preserved and undistorted; however there are numerous cracks, breaks, and holes present in elements. The first two teeth are present on both sides. Posteriorly, teeth are generally present on at least one side. Displacement of individual elements and fragments is typically minor and mostly confined to slight movement on the sagittal plane. Much of the right jugal-quadratojugal bar has been extensively physically reconstructed, showing little detail of its original morphology. There is a prominent break through the middle of the braincase and adductor chamber, separating the posteriormost quarter of the skull, visible roughly following the frontal-parietal contact on the skull roof. The plane of the break runs anterodorsally to posteroventrally. The cranium is sheared approximately 15° with the left side displaced anteriorly relative to the right. This is clearest seen in the shape of the braincase, the relative positions of the lacrimals, postorbitals, and the frontal-parietal contact (and associated break). The cranium has been laterally compressed (Balanoff *et al*., 2009). The posterior half is especially slender and the foramen magnum is tall and thin. It seems reasonable that lateral compression of the skull caused both the braincase to collapse in on itself and the strap-like and thin postorbital-squamosal and jugal-quadratojugal connections, and the plate-like quadrate-pterygoid flange to collapse medially, pivoting inwards posterior to where they are better supported mediolaterally in the orbital region by the skull roof via the frontal and the palate via the ectopterygoid.

The mandible of IVPP V 13326 (not CT-scanned) is preserved partially in two pieces; an anterior section comprising both rami extending posteriorly to just beyond the termination of the mandibular fenestra and a posterior piece representing the right articular surface and retroarticular process (unable to be located; potentially lost).

*Cranial retrodeformation procedure*

The IVPP V 13326 CT-dataset is the oldest and lowest resolution dataset used in this study. The images, though relatively low resolution, show good contrast between bone, teeth, and matrix, along with presumed neurovascular spaces in the premaxilla and braincase. The fossil bone and tooth material was segmented in Avizo Lite (version 9.3.0). Pulp cavities inside the teeth were also segmented. A large amount of elements required mirroring from the more complete right side to the left and/or repositioning. Individual elements/fragments were segmented and assigned into their own label fields. As the purpose was restoration (rather than osteological description) this was loosely focussed on individual bones, but more focussed on identifying ‘retrodeformational units’ that required repositioning/mirroring work.

To correct the deformational shear, all label fields were subjected to the Avizo ‘Shear’ module (setting 15 degrees); the CT-dataset was already aligned for this to work correctly. Label fields of those units that required mirroring were duplicated and the relevant axis reversed. Surface meshes generated from the label fields were then repositioned in the project window using the transform editor to restore the cranium as far as possible. Repositioning of cranial elements was done in relation to a base that consisted of the most complete part of the skull in which elements were correctly articulated. This consisted of the left premaxilla, right premaxilla (lower bigger part), right maxilla, right nasal (without nares fragment), left lacrimal, left jugal, left palatine, vomer, left pterygoid (+epipterygoid), left quadrate (lower bigger part), left quadratojugal (lower bigger part), parabasisphenoid, left postorbital, both frontals (and including parts of the orbitosphenoids and laterosphenoids), and all segmented teeth, including internal replacements.

Initially, another complex of respectively correctly positioned bones was repositioned onto this base. These were the posterior bones of the skull, comprising much of the braincase and adductor chamber, slightly displaced from the main base of skull bones by the prominent anterodorsal-posteroventral break. These comprised both parietals, supraoccipital, the lateral braincase elements of both sides, basioccipital, left quadrate (upper smaller part), left quadratojugal (upper smaller part), and left squamosal. The only other repositioned elements that were not mirror images of those from the more complete right side were a smaller upper part of the left premaxilla (?upper part of maxillary process) and a fragment forming the dorsal margin of the nares.

Those bones required to be mirrored, due to absence on the left side, were mirrored from the right and correctly positioned in the following units:

- A unit comprising the mirrored right quadrate (bigger lower part), right quadratojugal (bigger lower part), right pterygoid (including epipterygoid), right ectopterygoid, and the right palatine (left not used so the unit fit correctly as detailed below).
- A unit comprising the mirrored right squamosal, right quadrate (smaller upper part), right quadratojugal (smaller upper part).
- Mirrored right jugal.
- Mirrored posterior of the right parietal and right paroccipital process.
- Mirrored right portion of the parabasisphenoid to complete its missing left side.
- Mirrored teeth from right side, not present on left, to complete left tooth row.

Though all of the left palatine, all of the left nasal, much of left maxilla (though in two parts), much of the left postorbital, the medial half of left lacrimal, and anterior half of left pterygoid+epipterygoid, are actually preserved on the left side, fitting these bones to correctly contact their neighbours once their missing neighbours had been mirrored from the right side proved problematic, especially where forming complex contacts such as in the palate. These bones listed were therefore not used in the assembled model. Instead, mirrored duplicates of the right palatine, right nasal, right maxilla, right lacrimal, and right pterygoid+epipterygoid (which were all more complete anyway) were used and improved overall model quality.

The medial half of the articular surface of the quadrate was still missing (absent from the right quadrate and its mirrored element). To model the geometry of this small missing area without being too speculative, the corresponding region was scaled and added to the assembled *Incisivosaurus* skull from the quadrate of a photogrammetric 3D model of *Avimimus* specimen PIN 3907/3 (model produced in Agisoft Photoscan (version 1.3.4) using 107 photos from a Nikon D3300 DSLR camera with a 50mm lens). This seemed reasonable as the two species are early diverging oviraptorosaurians and have a similar quadrate morphology. It is also a very small region, likely of little importance in muscle reconstruction or finite element analyses (FEA), and essentially an aesthetic addition.

The repositioned component surfaces of the cranium were scanned back to label fields using the ‘Scan to Volume’ Avizo module and combined back into one label field using the ‘Relabel’ module. Cracks, breaks, and smaller missing areas were then corrected with the ‘paintbrush’ tool and interpolation using the segmentation editor (see Lautenschlager, 2016). During this step, some additional minor asymmetry was corrected in the premaxilla with the paint tool, the incisor-like teeth were repositioned with a slight rotation to make them more symmetrical (including their pulp cavities), minor asymmetry in the dorsal arch of the nares was corrected, and teeth 3 and 4 were slightly elongated to more resemble other examples of *Incisivosaurus* (STM22-6, previously assigned to *Similicaudipteryx*, now *I. gauthieri* (Xu, 2020).

The unsheared and restored skull, though now complete and usable for further analyses, is still likely deformed mediolaterally – the model is remarkably slender. Balanoff *et al*. (2009) remarked the endocranial cast of the skull to be extremely mediolaterally compressed, and the foramen magnum to be tall and slender. Other closely related early diverging oviraptorosaurians (such as *Avimimus portentosus* and *A. nemegtensis* (Funston *et al*., 2018)) possess very circular foramina magna, as do related theropods of similar size such as dromaeosaurids. To reduce the amount of mediolateral compression in the *Incisivosaurus* model with some degree of objectivity, the Avizo model surface was imported into Blender (version 2.90.0) and the postorbital region we identified as most prone to compression (see *Condition and taphonomic damage* section) was expanded laterally using a ‘Lattice’ deform modifier until the foramen magnum was of a similar height:width ratio (11:10) as *Avimimus portentosus* PIN 3907/3, also an early diverging oviraptorosaurian. At this stage, the jugal-quadratojugal bar (which in IVPP V 13326 is physically restored with matrix and cement) was also straightened out as it bends to be medially convex, likely erroneously obfuscating space for musculature. This final retrodeformed model was imported back into Avizo and, through the ‘Scan to Volume’ module, used to replace the posterior morphology of the model that was segmented into different materials (bone, teeth, pulp) so the model would be divided into these different materials for use in future FEA study.

*Mandibular retrodeformation procedure*

A 3D model of the anterior mandible piece of IVPP V 13326 was created through photogrammetry; 115 photos taken with a Nikon D7200 DSLR camera were processed in Agisoft PhotoScan (version 1.0.4.1847). The posterior piece was created in Blender (version 2.90.0) using box modelling (see Rahman and Lautenschlager, 2016), guided by images of the piece figured by Xu *et al*. (2002), as this piece was not observed in person and may be lost.

In Blender, the relatively undeformed dentary symphysis was positioned in the correct position for a closed jaw in relation to the cranium. The less complete left ramus of the mandible was removed and the right ramus was retrodeformed to follow the margin of the retrodeformed skull’s premaxilla, maxilla, and jugal, manipulating the mesh mediolaterally with the ‘Elastic Deform’ brush in Blender’s ‘Sculpt Mode’. The adjusted ramus was then mirrored to the left side. The box modelled posterior piece was also mirrored from right to left. The retrodeformed anterior section of the mandible and the two rear pieces, once positioned correctly with regard to each other and the cranium, were exported as PLY files and imported into Avizo.

These surfaces were converted to a single label field with the ‘Scan to Volume’ module and the missing sections between front and rear were interpolated in the segmentation editor. Minor holes and imperfections in the photogrammetric model were reconstructed with the ‘‘paintbrush’’ tool. Nine dentary teeth were created as a separate material in this label field in the correct places based on where the photogrammetric model showed their presence/sockets. The exact internal size of the teeth (the extent of their roots) couldn’t be ascertained as the photogrammetric model doesn’t include internal detail, so the rearmost nine teeth in the upper jaw were mirrored dorsoventrally and inserted into the dentary as a reasonable approximation.

Finally, the complete mandible model was transferred from Avizo to Blender and widened in the same way the cranium’s mediolateral compression was corrected, using a ‘Lattice’ deform modifier.

**2. *Citipati osmolskae* MPC-D 100/798**

*Provenance*

MPC-D 100/798 was discovered at the Ukhaa Tolgod locality of the Djadokhta Formation of Mongolia (Clark *et al*., 2001; Clark *et al*., 2002). The Djadokhta Formation dates to the Campanian, Late Cretaceous (Dashzeveg *et al*., 2005; Dingus *et al*., 2008; Hasegawa *et al*., 2009). The specimen comprises a nearly complete skeleton and is the holotype for *C. osmolskae*. MPC-D 100/798 was first described by Clark *et al*. (2001) in a rapid communication focussing almost solely on the cranium and mandible. *Khaan mckennai* was first described in the same publication.

*CT-Scanning*

The mandible, hyoid, stapes, and scleral ossicles were removed from the cranium ‘block’ prior to CT-scanning in 1997 for Clark *et al*. (2002). The CT-dataset used in this study includes only the cranium of MPC-D 100/798 and was generated at the University of Texas High-Resolution X-ray CT Facility for A. Balanoff in 2010. Scan parameters were 250 kV and 2.8 mA, yielding 371 JPG images of 1024 x 1024 pixel resolution. Slice spacing is 0.25 mm and x-,y-resolution is 0.196 mm. This dataset was used in a number of studies into palaeoneurology, and cranial and brain shape evolution (Balanoff *et al*., 2013; Balanoff *et al*., 2014; Balanoff *et al*., 2016; Balanoff *et al*., 2018).

*Taphonomic damage*

The MPC-D 100/978 cranium is remarkably well preserved. All cranial elements are present; it was the first oviraptorosaurian cranium described preserving the stapes and epipterygoid bones (Clarke *et al*., 2001). Few cranial bones show obvious taphonomic damage. Areas of matrix are left unprepared within the endocranial and pneumatic spaces and support the delicate jugal-quadratojugal bars, parasphenoid process, epipterygoids, paroccipital processes, and the interfenestral bars of the maxilla within the accessory antorbital fenestrae.

Breaks/holes are minor and present in: the left quadratojugal; left frontal just anterior to the supratemporal fenestra; the right frontal just anterior to the supratemporal fenestra and just anterior to the contact with the right postorbital; the middle of the ventral surface of the right pterygoid; and in the anterior of the ventral surface of the left pterygoid. Fragments are missing from the dorsal margin of the right orbit, the articular surface of the left quadrate, and from the left paroccipital process. There is a loose contact between the right postorbital and frontal. A small nook in the left jugal near the posteroventral corner of the orbit is identified as a possible pathology by Clark *et al*. (2002). The posterior half of the cranium has a slight left/right shear most visible in the relative positions of the supratemporal fenestrae, quadrates, and paroccipital processes – positioned relatively anteriorly on the left, posteriorly on the right.

*Cranial retrodeformation procedure*

The CT-dataset shows poor contrast in some areas between fossil and matrix, primarily around the parabasisphenoid, interior of the beak, and within pneumatic areas (where no internal bony struts can be seen). Bone material was segmented from the remaining matrix in Avizo Lite (version 9.3.0), additionally guided by notes and photographs from physically examined oviraptorosaurian specimens. Small cracks, break, holes, and loose contacts were corrected through interpolation in the Avizo segmentation editor (see Lautenschlager, 2016). The larger missing fragments of the right orbit and left quadrate and paraoccipital process were corrected with mirroring: the label field file was duplicated and relevant mirroring axis flipped in the crop editor so a mirror image selection could be made in the segmentation editor and transferred directly into the main model. The matrix filled pneumatic areas of the left nasal were too delicate and complex to be distinguished in CT, so the corresponding structure of the right (more thoroughly physically prepared) was also mirrored to replace them.

Final correction of the minor asymmetry (left/right shear in posterior half) was initially performed in Landmark, but it became clear Blender offered better results. Using Blender’s ‘Lattice’ modifier to adjust each side of the posterior half of the cranium, symmetry was restored to the positions of the paroccipital processes, supratemporal fenestrae, and postorbital bars.

*Mandibular retrodeformation procedure*

The mandible of *Citipati osmolskae* specimen MPC-D 100/798 is not included in the CT-dataset and was unable to be studied in person due to the COVID-19 pandemic. The mandible of the Dzamyn Khondt, oviraptorid MPC-D 100/42 (previously misidentified as *Oviraptor philoceratops*, likely *Citipati* sp.; Clark *et al*., 2002) had been studied in person and modelled photogrammetrically, so was modified to be used as a stand-in. The photogrammetric model was constructed in Agisoft Photoscan (version 1.3.4) and comprised 2 chunks (of 61 and 37 photos) from a Nikon D3300 DSLR camera with a 50mm lens.

The morphology of the MPC-D 100/42 mandible is similar to that of MPC-D100/978 but mediolaterally thinner and of a slightly different geometry in lateral view. The photogrammetric model of the MPC-D 100/42 mandible was exported to Blender and the ‘Lattice’ deform modifier was used to slightly alter its shape and scale to closely match reference images of the MPC-D 100/978 mandible. The result articulated and fit convincingly with the retrodeformed cranium. We are confident in using this altered mandible as a stand-in due to its many similarities with the *C. osmolskae* holotype mandible, the opportunities we had to study its bony morphology in person (muscle attachments sites), and the fact the cranium is the more important component in our method as the bony constraint of muscle size.

**3. *Khaan mckennai* MPC-D 100/973**

*Provenance*

MPC-D 100/973 was discovered at the Ukhaa Tolgod locality of the Djadokhta Formation of Mongolia (Clark *et al*., 2001; Balanoff and Norell, 2012), of Campanian, Late Cretaceous age (Dashzeveg *et al*., 2005; Dingus *et al*., 2008; Hasegawa *et al*., 2009). MPC-D 100/973 is one of three nearly complete skeletons referred to *K. mckennai* (along with MPC-D 100/1002) by Clark *et al*. (2001) when the species was first described and MPC-D 100/1127 designated the holotype. MPC-D 100/973 differs from MPC-D 100/1002 and MPC-D 100/1127 as its cranium and mandible have been freed from the rest of its skeleton (permitting CT-scanning). These other two specimens are borne intact on large sandstone slabs. Additional information on the provenance and preparation of MPC-D 100/973 can be found in Balanoff and Norell (2012).

*CT-Scanning*

MPC-D 100/973 was scanned at the University of Texas High-Resolution X-ray CT Facility for A. Balanoff in 2009. The specimen comprising cranium, mandible, hyoids, and an axial vertebra was scanned along the coronal axis in two passes and the slices were reassembled digitally. Scan parameters were 210 kV, 0.14 mA, yielding 912 JPG images of 1024 x 1024 pixel resolution. Slice spacing is 0.1637 mm and x-, y-resolution is 0.076 mm. The CT-dataset was initially used by Balanoff and Norell (2012) for osteological description of *K. mckennai* (endocranial cast subsequently used in Balanoff *et al*., 2013; Balanoff *et al*., 2014; Balanoff *et al*., 2016; Balanoff *et al*., 2018).

*Condition and taphonomic damage*

The scanned portion of MPC-D 100/973 containing the cranium and mandible (and hyoids and a cervical vertebra) is heavily matrix-bound – the fossil material is very fragmentary. It preserves essentially all cranial bones in articulation, though many are damaged, deformed, and/or slightly out of position. Most notably, the premaxilla-nasal arch is missing, as are significant portions of the maxillae, pterygoids, palatines, parabasisphenoid, and epipterygoids. The skull roof is heavily fragmented and crushed, obfuscating the pneumatic areas within. The cranium (and likely also mandible) is dorsoventrally compressed compared to MPC-D 100/1127 and MPC-D 100/1002 (Balanoff and Norell, 2012). This is most noticeable in the shape of the orbit and foramen magnum, and the lack of a rounded skull roof. Much of the dorsoventral compression appears to be associated with breaks in the nasal processes of the premaxilla, lacrimal bones, postorbital bars, parabasisphenoid and quadrates. The roof and posterior of the skull may have tilted ventrally, rotating around the break in the lacrimals, with the nasal processes of the premaxilla breaking anteriorly as the postorbital bars fractured and the quadrates (and associated elements) cracked posteriorly.

The mandible is complete apart from minor cracks and small missing areas (in the surangulars and angulars) and displacement of some of the medial elements (left splenial and angular). It is articulated with the cranium but displaced posteriorly, exacerbating an overbite. It has likely been dorsoventrally compressed similarly to the cranium.

*Cranial retrodeformation procedure*

Relatively poor contrast between fossil and matrix, and the heavily damaged and fragmented thin cranial bone, make this CT-dataset the most challenging of the four to work with. Individual cranial bones were not segmented, instead, elements or fragments that required repositioning/mirroring work were segmented as ‘retrodeformational units’ in Avizo Lite (version 9.3.0). The largest of the units, comprising the most complete and articulated series elements, included the posterior nasals, the frontals, parietals and majority of the brain case, the latero- and orbitosphenoids, the occipital region dorsal of the foramen magnum, the medial part of the exoccipitals and their contribution to the paroccipital processes, the dorsal part of the squamosals and postorbitals (the supratemporal bar), the dorsal part of the lacrimals, and the dorsal part of the nasal process of the premaxilla. This series of elements was used as a base to reposition the other displaced elements in relation to.

The only other articulated unit comprised mostly ventral elements including the rest of the premaxilla, the maxillae, right jugal, right quadratojugal, the parasphenoid rostrum, vomer, left ectopterygoid, left palatine, and left pterygoid. All other repositioned units were individual cranial elements or fragments thereof. These included four parts of the left jugal, two parts of the left postorbital (jugal process), the left quadratojugal, two parts of the right postorbital, the left quadrate (upper part missing), the right quadrate (upper part missing), part of the right quadrate flange, two fragments of the right paroccipital process, and the basioccipital and lower part of the braincase immediately within the foramen magnum. Additionally, mirrored duplicates of the left ectopterygoid, left palatine, and left pterygoid were created to replace their missing right counterparts.

Surfaces meshes were generated from separate label fields for each unit that required repositioning. The surface meshes were then repositioned in the Avizo project window using the transform editor to restore the cranium as far as possible, before the surfaces were scanned to volumes and relabelled back into a single label field.

The cranium still had a number of significantly missing areas. The missing regions of the occiput, paroccipital processes, quadrate flanges and squamosals were replaced through a mix of interpolation using the Avizo paint tool and deforming elements from these regions taken from *Conchoraptor gracilis* specimen MPC-D 100/3006, which is closely related, using the Avizo transform editor. These elements were scanned to volumes so the 3D information could be manipulated and made to fit in the segmentation editor. Similarly, more minor parts of the vomer, epipterygoids, and basisphenoid, were taken from *Citipati osmolskae* MPC-D 100/798, and edited to replace missing regions – as these elements were not well preserved in *C. gracilis* MPC-D 100/3006 either. The missing parts of the nasal process of the premaxilla were created using the ‘paintbrush’ tool and interpolation.

A surface was generated of the now essentially complete cranium and exported from Avizo into Blender as, though the repositioning of elements had somewhat increased the height of the dorsoventrally compressed cranium, the cranium was still not of a similar height:length ratio as MPC-D 100/1127 and MPC-D 100/1002 (Balanoff and Norell, 2012), likely indicating the effect of some more plastic deformation in addition to the now corrected displacement of elements. This was corrected in two steps.

The height:width measurements of the orbit of the almost retrodeformed MPC-D 100/973 were compared to MPC-D 100/1127 and MPC-D 100/1002; an increase in cranial height of 1.16x was required. This was achieved by transforming the retrodeformation in Blender, correcting the orbit proportions. Subsequently, the cranial roof was still slightly too flat, reducing overall cranial height, when compared to reference images of MPC-D 100/1127 and MPC-D 100/1002. This compression of the dorsal pneumatic cavities within the skull roof of MPC-D 100/973 was corrected by dragging the dorsal surface of the frontals and parietals higher, until the pneumatic space within was increased. This completed the retrodeformed geometry of the MPC-D 100/973 cranium, giving it a morphology much more similar to the other *K. mckennai* specimens.

*Mandibular retrodeformation procedure*

The CT-dataset of *Khaan mckennai* MPC-D 100/973 was the only CT-dataset studied substantially including a mandible, which was nearly complete. The retrodeformation of the mandible was fairly straightforward and mostly done in Avizo. The elements of the mandible were easier to segment separately than the cranial elements of this dataset, as they were more loosely articulated and closer to the specimen’s surface. The more complete right splenial was mirrored to replace the left, as were parts of the surface of the right articular. This was done by duplicating the label field, flipping its axes in the crop editor, and then selecting the relevant element/area in the segmentation editor, changing back to the unmirrored original label field where the selection could be moved/added in. The left angular was repositioned slightly, also in the segmentation editor. Minor areas of the surface of other bones were mirrored and cracks and breaks filled using the ‘paintbrush’ tool and interpolation in Avizo’s segmentation editor.

A surface was created and exported to Blender, where the ‘Lattice’ deform modifier was used to correct minor asymmetry. The height of the mandible was also increased by the same correction factor as cranium (1.16x) as the influence of plastic deformation in the form of dorsoventral compression was likely to be similar.

**4. *Conchoraptor gracilis***

**4.1 MPC-D 100/3006**

*Provenance*

MPC-D 100/3006 is from the Khulsan locality of the Baruungoyot Formation, Mongolia (Funston *et al*., 2018). The Baruungoyot Formation dates to upper Campanian-lower Maastrichtian age, overlying the Djadokhta Formation (Gradziński and Jerzykiewicz, 1974a, b; Fanti *et al*., 2012). The specimen comprises the posterior half of the cranium and a small posterior section of the left mandibular ramus, along with assorted postcranial material (Balanoff, 2011; Balanoff *et al*., 2014).

*CT-Scanning*

MPC-D 100/3006 was CT-scanned at Ohio University for A. Balanoff, yielding 995 DICOM images of 698 x 460 pixel resolution. The voxels are cubic with a resolution of 0.092 mm. The CT-dataset was initially used for endocranial description of *C. gracilis* (Balanoff *et al*., 2014; endocranial cast also used in Balanoff *et al*., 2013, Balanoff *et al*., 2016, and Balanoff *et al*., 2018).

*Condition and taphonomic damage*

The cranium (posterior half) is internally bound by matrix, supporting disarticulated elements. The mandibular fragment is unconnected. Missing anterior elements include the nasals, majority of lacrimals, maxillae, premaxillae, vomer, palatines, and the anterior of the ectopterygoids and pterygoids. All elements of the posterior half of the cranium are preserved (apart from areas of the epipterygoids and parabasisphenoid) but many are fractured into two or three pieces and/or disarticulated. This appears caused by a mediolateral compression that has fractured, displaced, rotated, and overlapped elements rather than deforming them plastically. This may be linked to the suggestion of Balanoff *et al*. (2014) that IGM 100/3006 was not fully mature skeletally as its braincase sutures are not completely fused.

The MPC-D 100/3006 specimen (and CT-scan) also includes a small posterior fragment of the left mandibular ramus and retroarticular process.

**4.2 ZPAL MgD-I/95**

*Provenance*

ZPAL Mg-D I/95 is from the Hermiin Tsav locality of the Baruungoyot Formation, Mongolia (Kundrát, 2007; Kundrát and Janáček, 2007), of upper Campanian-lower Maastrichtian age (Gradziński and Jerzykiewicz, 1974a, b; Fanti *et al*., 2012). The specimen comprises a nearly complete cranium and mandible (Osmólska, 1976).

*Photogrammetry*

The cranium is separated into four sections: the braincase and the right epipterygoid, squamosal, and quadrate; the premaxillae, maxillae, and anterior vomer; the right pterygoid, posterior vomer, and ectopterygoid; and the left pterygoid, ectopterygoid, and quadrate. Each section was photographed using a Nikon D3300 DSLR camera with a 50mm lens, borne on a tripod and triggered remotely, either moving the camera or the specimen itself. Photogrammetric models were generated in Agisoft Photoscan (version 1.3.4) by processing ‘chunks’ representing the specimen photographed positioned in different orientations which were then aligned and merged:

The braincase section was photographed by moving the camera. Three separate chunks were processed comprising 58, 58, and 54 photos.

The premaxilla section was photographed by moving the camera. Three chunks of 45, 41, and 39 photos were processed.

The right pterygoid section was photographed keeping the camera static, rotating the specimen on a turntable. Two chunks of 36 and 34 photos were processed.

The left pterygoid section was also photographed using a turntable. Two chunks of 38 and 37 photos were processed.

*Condition and taphonomic damage*

The cranium of ZPAL Mg-D I/95 was figured whole by Osmólska (1976) but is now in four parts (as described above). The overall morphology of the cranium is undistorted. It is missing parts of the maxillary and nasal processes of the premaxillae, lateral areas of the maxillae, the regions surrounding the choanae. It notably lacks the majority of the lacrimals and the elements of the temporal, postorbital, and jugal arches. The posterior of the parietals, supraoccipital, and most of the basicranium are also missing, and the left lateral wall of the braincase is heavily damaged (Kundrát, 2007; Kundrát and Janáček, 2007). Numerous other areas are damaged in minor ways, such as pitting in the cranial roof (though this may in fact be the damaged remnants of pneumatic fenestrae in the frontals; Funston, 2019). Minor areas of matrix are left within the premaxillae, maxillae, and in and around the braincase.

The mandible comprises the left dentary, disarticulated left splenial, and much of the right postdentary ramus (mostly the surangular and articular, the angular is essentially absent). The left dentary and right postdentary elements are held together by matrix in the correct relative position.

**4.3 *Conchoraptor*: MPC-D 100/3006 and ZPAL MgD-1/95 composite**

*Cranial retrodeformation procedure*

The MPC-D 100/3006 CT-dataset offers the greatest detail of the four used in this study. All elements (and fragments thereof) could be segmented separately in Avizo Lite (version 9.3.0) with relative confidence – useful as the main focus of retrodeforming this specimen was repositioning individual elements and fragments. Surfaces were generated for each separately segmented cranial element and exported to Blender where they were repositioned to articulate as properly as possible.

Mirrored duplicates were made of the right jugal, anterior of the right pterygoid, the two parts of right quadratojugal, left ventrolateral surface of basisphenoid (where pterygoids connect), to fill in for where these elements were missing on the other side. The right squamosal and right laterosphenoid were also mirrored, though they had counterparts on the left, as this resulted in a better overall skull articulation (there may have been some plastic deformation on their left counterparts). Similarly, minor edits using the ‘Elastic Deform’ brush in ‘Sculpt Mode’ were made on thinner areas of the squamosals and quadrate flanges to improve bone articulation. This resulted in an essentially completely retrodeformed posterior half of the skull, with only minor gaps between elements in places. Nevertheless, MPC-D 100/3006 doesn’t preserve any elements rostral to the frontals, jugals, and pterygoids.

These elements of *C. gracilis* were sourced from specimen ZPAL MgD-1/95 which was studied in person and modelled photogrammetrically. The 3D models of ZPAL MgD-1/95 were imported into Blender and positioned to articulate and overlay the retrodeformed MPC-D 100/3006 in the correct position. Measurements of ZPAL MgD-1/95 from Balanoff *et al*. (2014) suggest this specimen is very similar in size to MPC-D 100/3006. Observations presented here suggest it is slightly bigger, especially considering the damage to the occipital region of ZPAL MgD-1/95, and the supposed status of MPC-D 100/3006 as a sub-adult (Balanoff *et al.,* 2014). The retrodeformed MPC-D 100/3006 was scaled up 1.149x to articulate correctly with ZPAL MgD-1/95.

The composite specimen still lacked some material, chiefly much of the lacrimals, the anterior jugals, the ectopterygoids, palatines, vomer, and small details of the maxilla. These elements were imported into Blender from the 3D retrodeformed model of *Khaan* specimen MPC-D 100/973, and correctly positioned and modified with the ‘Elastic Deform’ brush in ‘Sculpt Mode’ to function as a replacement.

With the elements from MPC-D 100/3006, ZPAL MgD-1/95, and the modified *Khaan* retrodeformation all occupying the correct positions in Blender, all were exported separately back to Avizo. They were converted to label fields with the same reference field so they were occupying the same relative positions - active selections in the segmentation editor could therefore be directly transferred between the label fields. In this way, a final complete composite was created in the segmentation editor adding necessary sections from ZPAL MgD-1/95 and the modified *Khaan* elements to the main articulated section of MPC-D 100/3006; any minor missing sections or loose element connections were corrected using the Avizo ‘paintbrush’ segmentation tool and interpolation, or the Avizo selection grow function (Lautenschlager, 2016).

Minor asymmetries in the orientation of the quadrates and paroccipital processes of this final model were corrected in Blender using the ‘Lattice’ deform modifier.

This final retrodeformed specimen gives a very similar cranial length to that given by Osmólska (1976) for the intact ZPAL Mg-D I/95.

*Mandibular retrodeformation procedure*

The mandible fragment from ZPAL MgD-1/95 includes the anteriormost part of the dentary and a disarticulated left splenial of the left mandibular ramus, and the dorsal parts of the length of the surangular with a fairly complete articular region of the right mandibular ramus. The fragment was digitised with photogrammetry, using the same camera set-up as the ZPAL MgD-1/95 cranial material. The model was constructed from 31 photos in Agisoft Photoscan (version 1.3.4).

The fragment was overlaid with a mirrored duplicate and the matrix was removed to give a good overall morphology of the mandible. 3D material from the mandible of *Khaan* was deformed to the shape of the *Conchoraptor* mandible, using a ‘Lattice’ modifier, to fit the missing lateral areas of the surangular and angular, and fill in for the medial mandibular morphology, giving the rami a realistic thickness. Some sculpted shapes were used to complete any final gaps. These various components were combined with a Boolean (Union) modifier and the overall retrodeformed shape matched reference images of other *Conchoraptor* specimens (MPC-D 100/20 and MPC-D NatGeo.2018.036a) and fit the retrodeformed skull. The model also closely resembled a number of specimens identified as *Conchoraptor* *gracilis* among the collections of the MPC that have been recovered from poachers and not yet assigned specimen numbers.

**References**

Balanoff, A. Oviraptorosauria: morphology, phylogeny, and endocranial evolution. *Doctoral dissertation, Columbia University* (2011).

Balanoff, A. M. *et al*. Best practices for digitally constructing endocranial casts: examples from birds and their dinosaurian relatives. *J. Anat.* **229**, 173–190 (2016).

Balanoff, A. M., Bever, G. S. & Norell, M. a. Reconsidering the Avian Nature of the Oviraptorosaur Brain (Dinosauria: Theropoda). *PLoS One* **9**, e113559 (2014).

Balanoff, A. M., Bever, G. S., Rowe, T. B. & Norell, M. A. Evolutionary origins of the avian brain. *Nature* **501**, 93–96 (2013).

Balanoff, A. M. & Norell, M. A. Osteology of *Khaan mckennai* (Oviraptorosauria: Theropoda*). Bull. Am. Mus. Nat. Hist.* **372**, 1–77 (2012).

Balanoff, A. M., Norell, M. A., Hogan, A.V. & Bever, G. S. The endocranial cavity of oviraptorosaur dinosaurs and the increasingly complex, deep history of the avian brain. *Brain, Behav. Evol.* **91**, 125–135 (2018).

Balanoff, A., Xu, X., Kobayashi, Y., Matsufune, Y. & Norell, M. Cranial osteology of the theropod dinosaur *Incisivosaurus gauthieri* (Theropoda: Oviraptorosauria). *Am. Mus. Novit.* **3651**, 1–35 (2009).

Chang, S. C., Gao, K. Q., Zhou, C. F. & Jourdan, F. New chronostratigraphic constraints on the Yixian Formation with implications for the Jehol Biota. *Palaeogeogr. Palaeoclimatol. Palaeoecol.* **487**, 399–406. (2017).

Clark, J. M., Norell, M. A. & Barsbold, R. Two new oviraptorids (Theropoda: Oviraptorosauria), Upper Cretaceous Diadokhta Formation, Ukhaa Tolgod, Mongolia. *J. Vert. Paleont*. **21**, 209–213 (2001).

Clark, J. M., Norell, M. A. & Rowe, T. Cranial anatomy of *Citipati osmolskae* (Theropoda, Oviraptorosauria), and a reinterpretation of the holotype of *Oviraptor philoceratops*. *Am. Mus. Novit.* **3364**, 1–24 (2002).

Dashzeveg, D. *et al*. New stratigraphic subdivision, depositional environment, and age estimate for the Upper Cretaceous Djadokhta Formation, southern Ulan Nur Basin, Mongolia. *Am. Mus. Novit.* **3498**, 1–31 (2005).

Dingus, L. *et al*. The geology of Ukhaa Tolgod (Djadokhta Formation, Upper Cretaceous, Nemegt Basin, Mongolia). *Am. Mus. Novit.* **3616**, 1–40 (2008).

Fanti, F., Currie, P. J. & Badamgarav, D. New specimens of *Nemegtomaia* from the Baruungoyot and Nemegt Formations (Late Cretaceous) of Mongolia. *PLoS One* **7**, e31330 (2012).

Funston, G.F., 2019. Anatomy, systematics, and evolution of Oviraptorosauria (Dinosauria, Theropoda). *Doctoral dissertation,* *University of Alberta* (2019).

Funston, G. F., Mendonca, S. E., Currie, P. J. & Barsbold, R. Oviraptorosaur anatomy, diversity and ecology in the Nemegt Basin. *Palaeogeogr. Palaeoclimatol. Palaeoecol.* **494**, 101–120. (2018).

Gradziński, R. & T. Jerzykiewicz. Dinosaur and mammal-bearing aeolian and associated deposits of the Upper Cretaceous in the Gobi Desert (Mongolia). *Sed. Geol.* **12,** 249–278 (1974).

Gradziński, R., and T. Jerzykiewicz. Sedimentation of the Bayan Goryot Formation. In *Results of the Polish-Mongolian Palaeontological Expeditions* (ed Jaworowska, K.) 111–146 (Warsaw: Palaeontologia Polonica, 1974).

Hasegawa, H., Tada, R., Ichinnorov, N. & Minjin, C. Lithostratigraphy and depositional environments of the Upper Cretaceous Djadokhta Formation, Ulan Nuur basin, southern Mongolia, and its paleoclimatic implication. *J. As. Earth Sci.* **35**, 13–26 (2009).

Kundrát M. Avian-like attributes of a virtual brain model of the oviraptorid theropod *Conchoraptor gracilis*. *Naturwissenschaften* **94**, 499–504 (2007).

Kundrát, M. & Janáček, J. Cranial pneumatization and auditory perceptions of the oviraptorid dinosaur *Conchoraptor gracilis* (Theropoda, Maniraptora) from the Late Cretaceous of Mongolia. *Naturwissenschaften* **94**, 769–778 (2007).

Lautenschlager, S. Reconstructing the past: methods and techniques for the digital restoration of fossils. *R. Soc. Open Sci.* **3**, 160342 (2016).

Osmólska H. New light on the skull anatomy and systematic position of *Oviraptor philoceratops*. *Nature* **262**, 683–684 (1976).

Rahman, I. A. & Lautenschlager, S. Applications of three-dimensional box modeling to paleontological functional analysis. *Paleontol. Soc. Pap.* **22**, 119–132 (2016).

Swisher, C. C. III *et al*. Further Support for a Cretaceous age for the feathered-dinosaur beds of Liaoning, China: New 40Ar/39Ar dating of the Yixian and Tuchengzi Formations. *Chin. Sci. Bull*. **47**, 135–138 (2002).

Xu, X. Filamentous integuments in nonavialan theropods and their kin: advances and future perspectives for understanding the evolution of feathers. In *The evolution of feathers* (ed C. Foth, C. & Rauhut, O. W. M.) 67–78 (Springer, 2020).

Xu, X., Cheng, Y. N., Wang, X. L. and Chang, C. H. An unusual oviraptorosaurian dinosaur from China. *Nature*, **419**, 291–293 (2002).
